# Translating animal models of SARS-CoV-2 infection to vascular, neurological and gastrointestinal manifestations of COVID-19

**DOI:** 10.1242/dmm.052086

**Published:** 2025-04-08

**Authors:** James Chung, Julia Pierce, Craig Franklin, Rachel M. Olson, Alan R. Morrison, James Amos-Landgraf

**Affiliations:** ^1^ Department of Veterinary Pathobiology, University of Missouri, Columbia, MO 65211, USA; ^2^ Vascular Research Laboratory, Providence VA Medical Center, Providence, RI 02908, USA; ^3^ Department of Research, Ocean State Research Institute, Inc., Providence, RI 02908-4734, USA; ^4^ Department of Internal Medicine, Alpert Medical School of Brown University, Providence, RI 02908, USA; ^5^ Laboratory for Infectious Disease Research, University of Missouri, Columbia, MO 65211, USA

**Keywords:** COVID-19, Infectious disease, Animal models, Cardiovascular biology, Neurology, Gastroenterology

## Abstract

Since the emergence of severe acute respiratory syndrome coronavirus 2 (SARS-CoV-2) initiated a global pandemic resulting in an estimated 775 million infections with over 7 million deaths, it has become evident that COVID-19 is not solely a pulmonary disease. Emerging evidence has shown that, in a subset of patients, certain symptoms − including chest pain, stroke, anosmia, dysgeusia, diarrhea and abdominal pain – all indicate a role of vascular, neurological and gastrointestinal (GI) pathology in the disease process. Many of these disease processes persist long after the acute disease has been resolved, resulting in ‘long COVID’ or post-acute sequelae of COVID-19 (PASC). The molecular mechanisms underlying the acute and systemic conditions associated with COVID-19 remain incompletely defined. Appropriate animal models provide a method of understanding underlying disease mechanisms at the system level through the study of disease progression, tissue pathology, immune system response to the pathogen and behavioral responses. However, very few studies have addressed PASC and whether existing models hold promise for studying this challenging problem. Here, we review the current literature on cardiovascular, neurological and GI pathobiology caused by COVID-19 in patients, along with established animal models of the acute disease manifestations and their prospects for use in PASC studies. Our aim is to provide guidance for the selection of appropriate models in order to recapitulate certain aspects of the disease to enhance the translatability of mechanistic studies.

## Teaser

**Summary:** COVID-19 is a multi-organ system disease. This Review aims to provide guidance on selecting suitable animal models for specific disease aspects to improve translatability of studies.

## Introduction

The emergence of severe acute respiratory syndrome coronavirus 2 (SARS-CoV-2) in December of 2019 marked the onset of an unprecedented worldwide pandemic that has had lasting impact. SARS-CoV-2 rapidly spread from its origins in the city of Wuhan, China, to become a global public health threat. A member of the beta coronavirus family, SARS-CoV-2 was initially associated with severe respiratory symptoms, including pneumonia, fever and acute respiratory distress syndrome (ARDS) (Martinez-Salazar et al., 2022; Pal et al., 2020). SARS-CoV-2 infects cells via the angiotensin-converting enzyme 2 (ACE2) receptor by using the mechanism described in Box 1. The primary driver of case fatality with COVID-19 is thought to be the pulmonary damage resultant from infection followed by an associated systemic cytokine storm (Box 2) and sepsis (Bao et al., 2020; Ketcham et al., 2021). However, it quickly became evident that the clinical presentation can be highly variable and even long-term, affecting not only the respiratory system but also the vascular, olfactory, neurological and gastrointestinal systems (Ostergaard, 2021).

**Box 1. Mechanism of SARS-CoV-2 cellular uptake**

SARS-CoV-2 is characterized by its capacity for efficient human-to-human transmission, primarily via respiratory droplets. Viral entry into host cells is orchestrated through the interaction between the spike glycoprotein (S protein) of the virus and the primary viral receptor − angiotensin-converting enzyme 2 (ACE2) − located on the surface of target cells (Alexander et al., 2020). Prior to binding ACE2, the S protein of SARS-CoV-2 is primed by transmembrane serine protease 2 (TMPRSS2), which involves cleavage of the S2 site of the S protein (Hoffmann et al., 2020; Iwata-Yoshikawa et al., 2022). Once this priming has occurred, the receptor-binding domain of S protein is able to interact with ACE2. The subsequent fusion of virus envelope to cellular membranes, driven by the S2 subunit of the S protein, facilitates viral entry and infection of the host cell (Hoffmann et al., 2020; Iwata-Yoshikawa et al., 2022). Although this has been recognized as the primary mechanism of cellular uptake of the virus and despite most SARS-CoV-2 research has investigated this mechanism, some studies have also suggested that integrins mediate cellular entry of the virus with minimal ACE2 expression (Liu et al., 2022a).

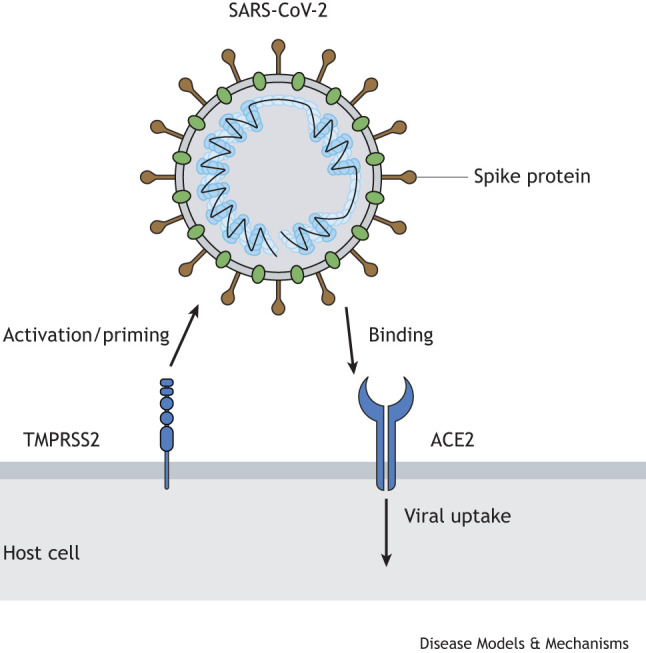

**Illustration of the primary cellular uptake mechanism of SARS-CoV-2.**

**Box 2. Glossary**

**Astrocytes:** Type of glial cell with multiple functions, including regulation of axonal growth and support, blood-brain barrier formation, higher cognition and memory.

**Bilateral lung infiltrates:** Presence of abnormal substances, such as fluid, cells or microorganisms, within the lung tissue, visible as shadows on a chest X-ray or CT scan.

**Capillaritis:** Inflammation of the capillary blood vessels, leading to blood leakage and pooling, commonly appearing as red or brown dots and patches on the skin.

**Cerebral vasculitis:** Inflammation of blood vessels within the brain, leading to narrowing or blockage, disruption of blood flow and damaging adjacent tissue. Characterized by inflammatory cell infiltration within the vessel wall.

**Chemokines:** Subset of cytokines that direct the migration of white blood cells to sites of inflammation.

**Choroid plexus:** Network of blood vessels located in the brain, responsible for producing cerebrospinal fluid and serving as the blood−cerebrospinal fluid barrier.

**Cytokine storm:** Dysregulated immune response leading to excessive release of pro-inflammatory cytokines, potentially resulting in multi-organ failure and death.

**Cytokines:** Protein signaling molecules released by immune and other cells to mediate and regulate immune system responses.

**Dyspnea:** Difficulty breathing or shortness of breath.

**Encephalitis/meningoencephalitis:** Inflammation of the brain due to infection or autoimmune response. Meningoencephalitis includes inflammation of the meninges, the membrane surrounding brain and spinal cord.

**Entorhinal cortex:** Located within the medial temporal lobe of the brain, connecting hippocampus and neocortex. Important for memory, navigation and perception of time.

**Hyaline membrane:** Thin membrane of dead cells and fluid lining the alveoli due to endothelial injury in the lungs, hindering gas exchange.

**Hyaline microthrombi:** Small eosinophilic structures composed of platelets and other proteins that are found in the lung capillaries and hinder gas exchange.

**Hypoxemia:** Low levels of oxygen in the blood.

**Infiltrates:** Cells or substances that are found in a non-typical location or at levels beyond those normally expected.

**Microglia:** Type of glial cell acting as the ‘immune cell of the brain’. Responsible for removing pathogens, dead cells and other harmful substances.

**Myocarditis:** Inflammation of the myocardium (the middle layer of the heart wall), which consists of striated muscle responsible for pumping the heart.

**Neuronal synapse:** Junction where neurons communicate via electrical or chemical signals.

**Neurophagia:** Destruction of neurons by microglial cells, histologically characterized by dying neurons surrounded by microglial cells.

**Olfactory trigone:** Brain area in which the olfactory tracts end; has a key role in the sense of smell.

**Peribronchiolar lymphocytic hyperplasia:** Proliferation of lymphocytes around bronchioles, occurring naturally with age or in response to systemic illnesses, such as infection.

**Perivascular cuffing:** Histological description of leukocytes surrounding a blood vessel; indicates leukocyte migration to the target tissue.

**Plethysmography:** Pulmonary function test measuring the volume of air moving in and out of the lungs.

**Satellitosis:** Increased number of cells surrounding a neuron; indicative of a neoplastic or reactive process.

**Suppurative:** Forming or discharging pus, typically consisting of necrotic debris, neutrophils and bacteria.

**Tachypnea:** Abnormally fast and shallow breathing.

**Thrombocytopenia:** Low blood platelet levels, potentially leading to bleeding disorders.

**Troponin:** Protein that is found primarily within the heart muscle. It is released into the bloodstream as a result of cardiac injury and used as a marker of such injury.

**Vacuolar degeneration:** Histological description of swollen cells expanded by fluid-filled sacs (vacuoles).

**Vascular congestion:** Enlargement of blood vessels due to increased blood flow and/or pressure.

**Vasoplegia:** Condition of low vascular resistance and blood pressure with normal or increased cardiac output, caused by uncontrolled vasodilation. Often occurs after cardiac surgery, sepsis, or cardiac injury.

The World Health Organization (WHO) declared ‘coronavirus disease 2019’ (COVID-19) a global pandemic in March of 2020 and, over the next three years, there would be over 7 million deaths worldwide attributed to the disease [see WHO, WHO COVID-19 dashboard: Number of COVID-19 cases reported to WHO (https://data.who.int/dashboards/covid19/cases?n=c, accessed 4 December 2023)]. Although the Public Health Emergency for COVID-19 was declared to be over in May 2023, the virus continues to evolve and appears to be transitioning towards endemicity, posing ongoing cyclical health risks worldwide. Reported hospital admissions and COVID-19 deaths in the USA have markedly declined compared to the early stages of the pandemic; however, the toll of COVID-19 on lives and long-term health consequences remains a sobering reality [see Centers for Disease Control and Prevention (CDC), National Center for Health Statistics: COVID Data Tracker (https://covid.cdc.gov/covid-data-tracker/#datatracker-home, accessed 22 January 2024)]. In addition to acute presentations, by January 2023, approximately one in ten Americans reported to experience post-acute sequelae of COVID-19 (PASC), also referred to as long COVID, with 27% of those affected noticing significant limitations in their day-to-day activities [see CDC (https://www.cdc.gov/nchs/covid19/pulse/long-covid.htm)].

While significant progress has been made in identifying treatment strategies for those with acute COVID-19, much of the literature is descriptive and gaps persist in understanding the mechanisms causing the highly variable disease presentations. Refining or expanding clinical treatment strategies and public health measures to combat this disease as it becomes endemic will require a deeper mechanistic understanding of the molecular disease processes. Adequate disease modeling is an important approach that may help to identify potential therapeutic targets, improving patient outcomes. Development of translational animal models of SARS-CoV-2 infection that accurately reflect certain aspects of human disease remains an important challenge for the field moving forward.

The major goals of this Review are to define some essential requirements of animal models for the study of vascular, neurological and gastrointestinal (GI) manifestations of COVID-19, and to assess the strengths and weaknesses of currently established models in these areas.

## Animal models of COVID-19

Experimental animal models are crucial for understanding disease mechanisms, and selecting the most appropriate species to effectively replicate the specific clinical disease aspects is important. Effective, translatable animal models should replicate human disease phenotypes, have molecular and physiological similarities to humans, be practical for research and, ultimately, facilitate the development of diagnostics, treatments and preventative strategies for human disease (Choudhary et al., 2022). While no single model meets all the listed criteria, the insights gained from any individual model species are vital for furthering our understanding of human disease. For understanding SARS-CoV-2, several animal models have been employed, including mice, ferrets, hamsters and non-human primates (NHPs), each of which offers unique advantages and insights into various facets of COVID-19 (de Melo et al., 2021; Kumari et al., 2021; Rutkai et al., 2022; Arce and Costoya, 2021; Becker et al., 2021) (Table 1).

**Table 1.** Manifestations of COVID-19 in different species

Species	Respiratory disease	Cardiovascular disease	Neurological disease	Gastrointestinal disease	Strengths	Limitations
**Human**	Cough, shortness of breath, chest discomfort and pneumonia are common clinical outcomes (Cascella et al., 2023; Stokes et al., 2020). Pneumonia-related tissue changes including edema, epithelial damage, capillaritis (Box 2) or enothelialitis and diffuse alveolar damage (DAD) (Bosmuller et al., 2021). Severe infection can result in ARDS, characterized by hypoxemia, difficulty breathing and bilateral lung infiltrates (Box 2) as seen on chest imaging (Selickman et al., 2022).	Vascular comorbidities (e.g. hypertension, coronary artery disease, diabetes, etc.) associated with more severe disease presentation and worse outcomes (Geca et al., 2022; Huang et al., 2020; Szarpak et al., 2022). Myocardial injury, myocarditis, acute coronary syndrome, acute myocardial infarction, cardiac arrhythmia, cardiac arrest, venous thromboembolic disease and heart failure are common manifestations (Adu-Amankwaah et al., 2021). Survivors continue to face cardiovascular risks (e.g. cerebrovascular disorders, dysrhythmias, ischemic and non-ischemic heart disease, pericarditis, myocarditis, heart failure, and thromboembolic disease), increased rates of cardiovascular disease, mortality, and rehospitalization (Xie et al., 2022; Ayoubkhani et al., 2021; Daugherty et al., 2021).	Acute neurological symptoms, including anosmia, dysgeusia, fatigue, myalgia and headache (Oosthuizen et al., 2021; Appelman et al., 2024). Few instances of encephalitis and/or cerebellitis (Huang et al., 2020; Elmakaty et al., 2022).	Reported GI symptoms include diarrhea, abdominal pain and nausea (de Oliveira et al., 2020; Jin et al., 2020; Mao et al., 2020b; Pan et al., 2020; Sultan et al., 2020; Carnevale et al., 2021; Din et al., 2021). Intestine might be an extrapulmonary site of replication (Chen et al., 2020; Zuo et al., 2020). SARS-CoV-2 infection alters normal gut microbiome (Gu et al., 2020; Yeoh et al., 2021).	−	−
**hACE2-K18 mouse**	Severe pneumonia characterized by alveolar necrosis, edema, hemorrhage, fibrin deposition, alveolar and interstitial infiltrates (primary neutrophils and macrophages), and vasculitis with thrombosis (Oladunni et al., 2020; Arce and Costoya, 2021; Yinda et al., 2021).	n/a	Intranasal inoculation with USA-WA-01 SARS-CoV-2 resulting in fatal CNS infection is circumvented with aerosol inoculation (Fumagalli et al., 2022). Neurological symptoms (anosmia, seizures, tremors, proprioceptive deficits and ataxia) (Ye et al., 2021; Vidal et al., 2022).	High-dose infection leads to increased disruption of microbial diversity and richness in the cecum (Seibert et al., 2021).	Potential model for pulmonary, neurological and GI disease.	Fatal CNS infections observed in this mouse model may confound clinical data associated with acute infection, therefore results must be interpreted cautiously (Oladunni et al., 2020). Non-physiological hACE2 levels (Oladunni et al., 2020). No evidence of cardiovascular disease.
**HFH4-hACE2 mouse**	Noticeable respiratory distress and lethality in <50% of challenged mice (Jiang et al., 2020; Dinnon et al., 2020). Whole-body plethysmography indicated normal respiratory function (Dinnon et al., 2020).	Edema and necrosis observed in some cardiomyocytes (Jiang et al., 2020; Dinnon et al., 2020).	Intranasal inoculation with USA-WA-01 SARS-CoV-2 results in fatal CNS infection (Dinnon et al., 2020).	n/a	Potential model for pulmonary, cardiovascular and neurological disease.	Non-physiological hACE2 levels (Dinnon et al., 2020). CNS infection driver of mortality (Dinnon et al., 2020). No evidence of GI disease (Dinnon et al., 2020).
**SARS-CoV-2 MA10-infected mouse**	Viral replication shown in lungs and plethysmography showed changes indicative of airway obstruction (Dinnon et al., 2020; Leist et al., 2020). Diffuse alveolar damage, exfoliated cells in small airways, fibrin deposition, loss of surfactant, inflammatory infiltrates, peribronchiolar lymphocytic hyperplasia, vascular congestion and bronchial epithelial damage (Dinnon et al., 2020; Leist et al., 2020).	n/a	SARS-CoV-2 inoculation results in decreased expression of claudin-5 mRNA and increases levels of IBA1-positive microglial cells (Amruta et al., 2023). Infection induced neuroinflammation and neuropathogenesis without occurrence of fatal encephalitis (Amruta et al., 2023).	n/a	C57BL/6, BALB/c, and *Rag2^−/−^* mice on C57BL/6 background are susceptible to intranasal inoculation (Amruta et al., 2023). Potential model for pulmonary and neurological disease.	No neurological clinical signs noted (Amruta et al., 2023). Infection of C57BL/6 mice shows minimal clinical and histological evidence of disease (Dinnon et al., 2020; Leist et al., 2020). Inability to study variants. No evidence of cardiovascular or GI disease.
**Syrian hamster**	Viral replication in upper and lower respiratory tracts (Choudhary et al., 2022; Becker et al., 2021; Osterrieder et al., 2020; Imai et al., 2020). Lung abnormalities including inflammatory cell presence in airways, alveoli and interstitial spaces, and epithelial hypertrophy (Choudhary et al., 2022; Becker et al., 2021; Osterrieder et al., 2020; Imai et al., 2020).	Occurrence of vascular lesions and cardiac injury, including ventricular hypertrophy, ventricular wall thickening, increased coronary artery inflammation, interstitial coronary fibrosis, microthrombi and intracardiac platelet/fibrin aggregates (Francis et al., 2021; Rizvi et al., 2022; Xue et al., 2022). Observations of elevated levels of serum cardiac troponin I, cholesterol, HDL, LDL, VLDL and long-chain fatty acid triglycerides (Rizvi et al., 2022).	Transient anosmia and ageusia (Becker et al., 2021; Monchatre-Leroy et al., 2021; Käufer et al., 2022). Transient, non-productive CNS infection (Bryche et al., 2020; Monchatre-Leroy et al., 2021; Käufer et al., 2022).	No evidence of GI symptoms, but evidence of shift in gut microbiome composition after infection, correlating with disease severity and inflammation (Seibert et al., 2022; Sencio et al., 2022).	No diffuse CNS infection and disease (Bryche et al., 2020; de Melo et al., 2021). Natural susceptibility. Potential model for pulmonary, cardiovascular, neurological and GI disease.	Limited neurological clinical signs (Xydakis et al., 2020; Golden et al., 2020; Carpenter et al., 2023; Bryche et al., 2020; de Melo et al., 2021; Käufer et al., 2022). Limited resources (e.g. sequencing databases, antibodies, primers and other species-specific tools) compared to other animals.
**Ferret**	Detectable virus within upper respiratory tract, especially the nasal cavity (Au et al., 2022; Ciurkiewicz et al., 2022). Infectious virus not recovered from lungs; viral antigen not observed in the lungs (Everett et al., 2021; Au et al., 2022; Ciurkiewicz et al., 2022).	n/a	Observation of viral RNA in areas of brain (Schlottau et al., 2020; Everett et al., 2021; Au et al., 2022; Ciurkiewicz et al., 2022).	n/a	Good model for transmission studies (Richard et al., 2020; Kutter et al., 2021; Kim et al., 2020). Potential model for pulmonary disease.	No evidence of SARS-CoV-2-associated neurological signs or neuropathy (Everett et al., 2021; van de Ven et al., 2021; Au et al., 2022; Ciurkiewicz et al., 2022). No evidence of cardiovascular or GI disease (Everett et al., 2021; Ciurkiewicz et al., 2022).
**African green monkey**	Pulmonary consolidation, hyperemia, lesions of the lower lobe, terminal bronchiole inflammation and increased alveolar macrophages – all seen in histology of the lung (Hartman et al., 2020; Woolsey et al., 2021; Cross et al., 2020; Blair et al., 2021). Development of ARDS in aged animals, leading to fatality (Blair et al., 2021). Lethal cases experienced respiratory distress and evidence of SIRS, including dyspnea (Box 2), tachypnea (Box 2), hypothermia, reduced oxygen saturation, increased cytokine levels, and bronchointerstitial pneumonia (Blair et al., 2021).	Observations of thrombocytopenia (Box 2) and elevated C-reactive protein together with decrease in lymphocytes and platelets, and increase in neutrophils within the blood (Woolsey et al., 2021; Hartman et al., 2020).	Evidence of neuropathology and neuroinflammation (Rutkai et al., 2022).	n/a	Inflammatory response similar to that in humans. High genetic similarity to humans. Potential model for pulmonary, cardiovascular and neurological disease.	Ethical and safety concerns. Cost. Less common and available compared to other NHP models. No evidence of GI disease.
**Rhesus macaque**	Detection of viral RNA in lungs and nasopharyngeal swabs (Choudhary et al., 2022; Zheng et al., 2020; Shan et al., 2020; Speranza et al., 2022). Variable presentation of pneumonia (generally present in inferior lobes) with edema, hemorrhage, necrosis, alveolar wall thickening, endothelial damage, fibrosis and immune cell infiltration (Choudhary et al., 2022; Shan et al., 2020; Speranza et al., 2022).	Myocarditis, microthombi, increased coagulation, cardiomyocyte disarray and necrosis (Feng et al., 2021; Rabbani et al., 2022).	Evidence of neuropathology and neuroinflammation (microglial cell and astrocyte activation) (Jiao et al., 2021; Beckman et al., 2022; Philippens et al., 2022; Rutkai et al., 2022).	Transient diarrhea with alterations in composition of fecal microbiota (Sokol et al., 2021).	High genetic similarity to humans. Potential model for pulmonary, cardiovascular, neurological and GI disease.	Ethical and safety concerns. Cost.
**Cynomolgus macaque**	Pneumonia confirmed by computed tomography (Urano et al., 2021). Elderly animals (23−30 years old) showed longer periods of viral RNA detection and longer periods of pneumonia (including recurrence) (Urano et al., 2021). Presence of alveolar necrosis, thickened alveolar walls, generation and sloughing of bronchiolar epithelium and hyperemia in caudal pulmonary lobes (Urano et al., 2021; Salguero et al., 2021; Boszormenyi et al., 2021).	Detectable virus in heart tissue of elderly animal (23−30 years) (Urano et al., 2021).	Evidence of neuropathology and neuroinflammation (microglial cell activation) (Philippens et al., 2022).	Alterations in composition of fecal microbiota (Sokol et al., 2021).	High genetic similarity to humans. Potential model for pulmonary, cardiovascular, neurological and GI disease.	Ethical and safety concerns. Cost.

**Abbreviations:** (h)ACE2, (human) angiotensin-converting enzyme 2; ARDS, acute respiratory distress syndrome; CNS, central nervous system; GI, gastrointestinal; HDL, high-density lipoprotein; LDL, low-density lipoprotein; VLDL, low-density lipoprotein; SARS-CoV-2 MA10, mouse-adapted strain of SARS-CoV-2; SIRS, systemic inflammatory response syndrome.

Rodents, such as mice and rats, are frequently employed in translational studies due to their short life cycles, cost-effectiveness, physiological and genetic similarities to humans, and relative ease of genetic manipulation, making them valuable models of various human diseases, including COVID-19 (Bryda, 2013; Mouse Genome Sequencing Consortium, 2002). Standard inbred strains of mice, such as BALB/c and C57BL/6, initially posed challenges due to the inefficiency of SARS-CoV-2 binding to murine ACE2 receptors (Zhao et al., 2020). Infection of these mice with SARS-CoV-2 revealed no overt clinical manifestations, weight loss or mortality (Mohandas et al., 2020). Viral presence appeared contained to the lungs and was rapidly cleared (Mohandas et al., 2020). However, subsequent variants of concern (alpha, omicron, etc.) have shown evidence of binding to mouse ACE2 (Chen et al., 2022; Fumagalli et al., 2022). Two strategies have been employed to address this major limitation of murine models: 1) the generation of mice that transgenically express human ACE2 protein (Fumagalli et al., 2022) and, 2) the use of selectively adapted viral strains that have higher affinity for mouse ACE2 (Dinnon et al., 2020). Apart from mice, Syrian hamsters are also a commonly used rodent model for viral infections. These animals are naturally susceptible to SARS-CoV-2, eliminating the need for genetic engineering (de Melo et al., 2021).

Beyond rodents, ferrets are often used in respiratory disease research as their respiratory tract anatomy and physiology is comparable to humans (Belser et al., 2011). They have natural susceptibility to human viral pathogens, most notably influenza virus (van Riel et al., 2007). NHPs, such as rhesus and cynomolgus macaques, are also valuable for studying complex diseases and when testing potential vaccines or treatments due to their close genetic and physiological similarity to humans (Vallender and Miller, 2013). Despite limitations, such as specialized husbandry needs, longer lifespans and more ethical considerations, non-rodent models remain an indispensable aspect of pre-clinical research where rodent models fall short.

**Fig. 1.**
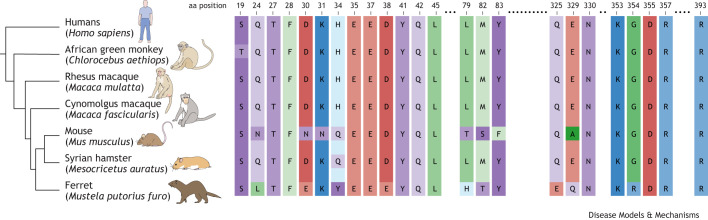
**Key amino acid (aa) sequences that determine the protein structure of ACE2 lead to differential susceptibility of SARS-COV-2 infection**. Shown are conserved aa sequences of ACE2 for seven species with varying susceptibility to infection with SARS-CoV-2. Species with ACE2 sequences most similar to that in human show a natural susceptibility to SARS-CoV-2 infection; they include African green monkey, rhesus macaque, cynomolgus macaque, Syrian hamster and ferret. Of these seven species, mouse is the only one that does not show natural susceptibility and requires transgenic models. Notably, mice exhibit many key differences regarding these aa, including deviations from a certain aa category (e.g. a hydrophobic aa in the human sequence is replaced with a polar/hydrophilic aa in the mouse sequence). These types of alteration may contribute to the reduced infectibility of mice. Hydrophobic aa phenylalanine, glycine, leucine and methionine (F, G, L and M, respectively) are shown in green; polar aa asparagine, serine, glutamine, threonine and tyrosine (N, S, Q, T and Y, respectively) are shown in purple; basic aa histidine, lysine and arginine (H, K and R, respectively) are shown in blue; acidic aa aspartate and glutamate (D and E, respectively) are shown in red. Adapted with permission from Alexander et al. (2020).

The varying natural susceptibility to SARS-CoV-2 of these animal models can be explained by differences in their ACE2 protein sequences compared to that of human ACE2 (Alexander et al., 2020). Species with ACE2 sequences more similar to the human one − especially in key conserved regions − are more likely to be susceptible to SARS-CoV-2 infection than those with less similar ACE2 (Alexander et al., 2020). Key amino acids (aa) of the ACE2 protein, and differences among different animal models and ACE2 in humans, are illustrated in Fig. 1.

## Respiratory, pulmonary and vascular disease

### Human disease manifestations

In most patients, SARS-CoV-2 infection begins with the inhalation of virus-laden respiratory droplets. COVID-19 presents with a wide range of respiratory symptoms that vary in severity. While some individuals may experience mild symptoms or remain asymptomatic, others develop more severe respiratory distress. Common respiratory manifestations include cough, shortness of breath and chest discomfort (Cascella et al., 2023; Stokes et al., 2020). Pneumonia is a frequent complication and a significant contributor to respiratory distress in patients (Stokes et al., 2020; Cascella et al., 2023). Observed tissue changes are listed in Table 1. In severe cases, COVID-19 can lead to ARDS, characterized by profound hypoxemia (Box 2), difficulty breathing and bilateral lung infiltrates (Box 2) on chest imaging (Selickman et al., 2022).

Increasing evidence suggests that COVID-19 is more than just a respiratory disease, with some experts classifying it as a vascular disorder (Adu-Amankwaah et al., 2021; Liu et al., 2021; Siddiqi et al., 2021). Individuals with pre-existing vascular comorbidities, such as hypertension, coronary artery disease and diabetes, often experience more severe disease and worse outcomes when infected with SARS-CoV-2 (Geca et al., 2022; Huang et al., 2020; Szarpak et al., 2022). Pre-existing heart failure is associated with an almost 50% increased mortality, and patients with cardiovascular disease (CVD) and increased levels of troponin (Box 2) have been found to be associated with a 10% increase in case fatality ratio (CFR) compared with patients without such comorbidities (Bhatt et al., 2021; Dan et al., 2020). A growing body of literature recognizes vascular injury and resulting dysfunction as a crucial mechanism underlying COVID-19 clinical manifestations (Connors and Levy, 2020; Iba et al., 2020; Levi et al., 2020; Liu et al., 2021). This vascular involvement in COVID-19 pathogenesis underscores the importance of understanding and modeling these aspects of the disease.

**Fig. 2.**
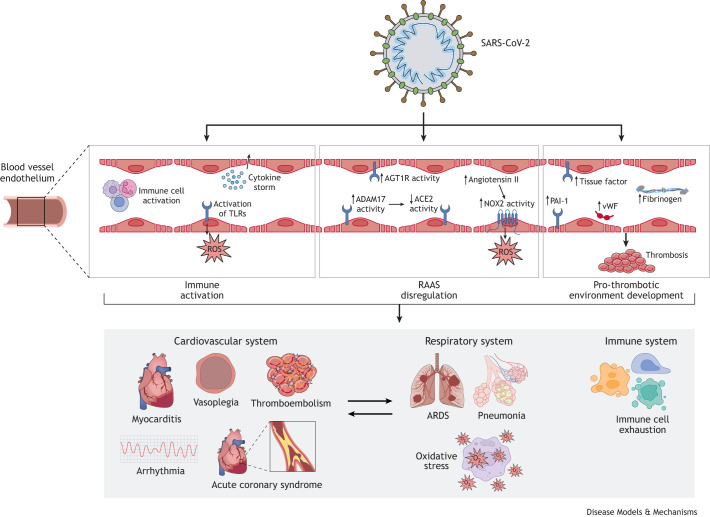
**Vascular manifestations of SARS-CoV-2 infection.** Illustration of the three primary vascular pathways impacted during systemic manifestations of SARS-CoV-2 infection. The impact of SARS-CoV-2 on these pathways may be consequent to direct or indirect injury of vascular endothelial cells. Following infection, immune activation, disruption of the renin-angiotensin-aldosterone system (RAAS) and promotion of a pro-thrombotic environment all contribute to complications of the cardiovascular, respiratory and immune system. Mechanisms of immune activation include cytokine storm, immune cell activation and activation of toll-like receptors (TLRs) with subsequent increased production of reactive oxygen species (ROS). RAAS disruption can also occur through multiple pathways. Notably, increased activity of A Disintegrin and Metalloproteinase 17 (ADAM17), which is responsible for cleavage of the ACE2 membrane-bound receptor, results in decreased membrane ACE2 activity and dysregulation of the RAAS. Increased activity of angiotensin II receptor type 1 (AGT1R) contributes to this dysregulation and promotes oxidative stress, apoptosis and fibrosis. Upregulation in the levels of angiotensin II, a cleavage product of angiotensinogen, also contributes to the production of NADPH oxidase 2 (NOX2) that, in turn, results in ROS production. Upregulation and overexpression of multiple markers can also result in a pro-thrombotic and hypercoagulation environment within the endothelium and result in thrombosis. Increased protein levels of tissue factor (*F3*), plasminogen activator inhibitor-1 (PAI-1), von Willebrand factor (vWF) and the glycoprotein complex fibrinogen all contribute to this pro-thrombotic environment. The consequences of immune activation, dysregulation of the RAAS and development of a pro-thrombotic environment within the endothelium affect the cardiovascular system, respiratory system and immune system. Within the cardiovascular system, it can result in clinical manifestations including myocarditis, vasoplegia, thromboembolism, arrhythmia and acute coronary syndrome. Respiratory complications may include acute respiratory distress syndrome (ARDS), pneumonia or oxidative stress. The crucial relationship between the cardiovascular system and respiratory system also means that development of these symptoms within one system may influence the likelihood of developing disease of the other system. These pathways can also cause immune exhaustion, leading to a reduced ability to fight infection.

Cardiovascular impacts of SARS-CoV-2 infection have been well-documented. A study conducted in Wuhan, China in early 2020 found that nearly 20% of hospitalized patients developed cardiac injury (Shi et al., 2020). These patients experienced more complications and had a higher mortality rate (51.2% vs 4.5%, *P*<0.001), even after accounting for age and pre-existing conditions (Shi et al., 2020). During the acute phase of infection, clinical reports have documented various common manifestations of cardiovascular injury (Table 1) (Adu-Amankwaah et al., 2021). The exact mechanisms remain unclear, but current reports suggest that endothelial infection play a central role. Endothelial infection may have multiple effects, including (i) disruption of the renin-angiotensin-aldosterone system (Cojocaru et al., 2023; Violi et al., 2020; Zipeto et al., 2020), which has been implicated as a potential mediator of the relationship between cardiovascular disease and COVID-19 severity (Coto et al., 2021); (ii) immune system activation (Giamarellos-Bourboulis et al., 2020); (iii) increased endothelial permeability, with resulting vascular leakage (Joffre et al., 2022) and, (iv) development of a pro-thrombotic environment (Fig. 2) (Adu-Amankwaah et al., 2021; Siddiqi et al., 2021; Nguyen et al., 2022). Endothelial injury can be further compounded by activation of toll-like receptors (TLRs) (Mantovani et al., 2023), resulting in increased production of reactive oxygen species (ROS) (Siddiqi et al., 2021; To et al., 2017). All of these contribute to symptoms such as ARDS, myocardial injury, thromboembolism and vasoplegia (Box 2) (Siddiqi et al., 2021; Adu-Amankwaah et al., 2021).

Beyond the acute phase of infection, COVID-19 survivors continue to face cardiovascular risks, including cerebrovascular disorders, heart disease and heart failure (Table 1) (Xie et al., 2022). Several studies have shown similarly increased rates of cardiovascular disease, post-discharge mortality and rehospitalization of initially hospitalized patients (Ayoubkhani et al., 2021; Daugherty et al., 2021). Patients hospitalized for COVID-19 infection who had not been admitted to the ICU had three times higher rates of cardiovascular disease, eight times higher rates of mortality and four times higher rates of rehospitalization compared with people not diagnosed with COVID-19 (Ayoubkhani et al., 2021). There appears to be a reciprocal relationship between CVD and COVID-19 infection by which CVD serves as a risk factor for and increases the severity of infection, and by which COVID-19 infection serves as a risk factor for and increases in severity of CVD.

### Rodent models

The use of rodent models in studying the respiratory, pulmonary and vascular aspects of COVID-19 has provided valuable insights. The B6.Cg-Tg(K18-hACE2)2Prlmn/J (also known as K18-hACE2) mouse is one of the best-established transgenic mouse models for studying SARS-CoV-2 (Oladunni et al., 2020; Arce and Costoya, 2021; Yinda et al., 2021; Dong et al., 2022). This mouse line has originally been developed in 2006 as a model for studying the original SARS-CoV, a related coronavirus with >70% homology to SARS-CoV-2 (McCray et al., 2007). These mice express the human *ACE2* gene (and, subsequently, the human ACE2 protein) under the control of the keratin 18 (*KRT18*; hereafter K18) promoter. This leads to expression in epithelial cells − including those of the airway − but may also enable expression in the central nervous system (CNS), GI system and kidneys (Laboratory, 2023; Carossino et al., 2022; Rabbani et al., 2022). Following intranasal challenge with SARS-CoV-2, these mice developed severe pneumonia characterized by manifestations detailed in Table 1 (Oladunni et al., 2020; Arce and Costoya, 2021; Yinda et al., 2021). This mouse model exhibited elevated levels of local and systemic chemokines (Box 2) and cytokines (Box 2), similar to the cytokine storm of human patients with COVID-19 (Ye et al., 2020; Wang and Ma, 2008; Parsons et al., 2005). K18-hACE2 mice are considered to be an ACE2-overexpression model and infection of these mice with SARS-CoV-2 can be highly lethal, making them an attractive model for acute severe disease (Yinda et al., 2021). There also appear to be minimal acute cardiac or vascular disease complications, probably due to the lack of K18 promoter-driven hACE2 expression in the heart and endothelium, but long-term effects merit further study (Yinda et al., 2021).

Another transgenic model, the Hfh4-ACE2 mouse, was developed to study W1V1-CoV, a SARS-like virus (Dinnon et al., 2020; Menachery et al., 2016; Jiang et al., 2020). Under the control of the forkhead box J1 (*FOXJ1*; hereafter HFH4/FOXJ1) promoter, these mice express human ACE2 primarily in lung ciliated epithelial cells, but expression has also been noted in the brain, liver, kidney and GI tract (Menachery et al., 2016). When infected with SARS-CoV-2, these mice display clinical signs in two distinct clusters, i.e. <50% showed significant weight loss, noticeable respiratory distress and neurological symptoms that progressed to lethality, whereas the remaining mice stayed asymptomatic and survived acute challenge (Dinnon et al., 2020; Jiang et al., 2020). Edema and necrosis were observed in some cardiomyocytes, while whole-body plethysmography (Box 2) indicated normal respiratory function (Dinnon et al., 2020; Jiang et al., 2020), suggesting − as it does not seem to replicate human disease − that this mouse model is less suitable for studying respiratory manifestations.

In contrast, the mouse-adapted strain of SARS-CoV-2 (SARS-CoV-2 MA10) involved passaging SARS-CoV-2 ten times in BALB/cAnNHsd (BALB/c) mice to enable infection of mice that express the native murine ACE2 receptor (Dinnon et al., 2020; Leist et al., 2020). This approach has shown promise in modeling human-like disease presentations in certain mouse strains, such as BALB/c (Dinnon et al., 2020; Leist et al., 2020). Young mice showed little to no overt clinical signs of infection, but viral replication was supported in lungs, and plethysmography showed changes indicative of airway obstruction and altered exhalation flow rate (Dinnon et al., 2020; Leist et al., 2020). Older (1-year-old) mice showed increased mortality, localized changes in cytokine and chemokine levels, and a more severe disease presentation (Dinnon et al., 2020; Leist et al., 2020). Infection of young (10-week-old) mice at higher challenge doses also led to lethality (Leist et al., 2020). Histological changes were consistent with those observed for COVID-19 in humans and included diffuse alveolar damage, exfoliated cells in small airways, fibrin deposition, loss of surfactant, inflammatory infiltrates, peribronchiolar lymphocytic hyperplasia (Box 2), vascular congestion (Box 2) and bronchial epithelial damage (Dinnon et al., 2020; Leist et al., 2020). In contrast, infection of C57BL/6 mice demonstrated lower clinical and histological scores, and reduced viral replication, as well as quicker restoration of lung function (Leist et al., 2020). A limitation in this model is that human variants of concern (VOC) often involve mutations of the spike protein, rendering a mouse-adapted virus less suitable for studying viral evolution, transmission and associated varying disease manifestations.

In wild-type Syrian hamsters, infection led to viral replication in both the upper and lower respiratory tracts, with peak viral loads at ∼3 days post challenge (Choudhary et al., 2022; Becker et al., 2021; Osterrieder et al., 2020; Imai et al., 2020). They exhibited lung abnormalities including inflammatory cells in the airways, alveoli and interstitial spaces, along with epithelial hypertrophy, with vascular lesions and cardiac injury additionally noted (Table 1) (Francis et al., 2021; Rizvi et al., 2022; Xue et al., 2022). These hamsters showed elevated levels of various molecular markers of cardiac disease, including cholesterol and triglycerides (Table 1) (Rizvi et al., 2022). This animal model also showed age-dependent differences, with younger hamsters experiencing a more robust immune response, reduced clinical severity and faster recovery (Osterrieder et al., 2020).

Although wild-type Syrian hamsters are more commonly used in SARS-CoV-2 research, COVID-19 modeling has also included Roborovski dwarf hamsters and transgenic hACE2-expressing hamsters. Following infection, dwarf hamsters developed severe clinical signs and evidence of diffuse alveolar damage and hyaline microthrombi (Box 2) in the lungs (Trimpert et al., 2020). K18-hACE2 hamsters, expressing human ACE2 under the control of the same *KRT18* promoter as the K18-hACE2 mice, were susceptible to severe lethal disease (Golden et al., 2022). Weight loss began as early as day 2 post challenge and a large proportion succumbed to disease by day 5 (Golden et al., 2022). The infection resulted in mild pulmonary lesions but severe nasal cavity and CNS lesions, which will be discussed below.

The natural susceptibility of some hamster models to SARS-CoV-2 make them appealing models. One critical limitation to the use of hamsters compared with murine models, is the limited availability of research tools like sequencing databases, antibodies, primers, and other hamster-specific cellular and molecular probes (Miao et al., 2019). This may limit the breadth of studies that can be conducted with hamster models (Table 1).

### Non-rodent models

Ferrets, analogous to hamsters, have ACE2 receptors that are naturally susceptible to SARS-CoV-2 binding, although the binding affinity is considered low, especially in the lower respiratory tract (Everett et al., 2021). Infection of ferrets with SARS-CoV-2 primarily impacts the respiratory tract, and both intranasal and intratracheal exposure results in productive infections (Everett et al., 2021; van de Ven et al., 2021). The highest viral loads are typically found in the upper respiratory tract, especially in the nasal cavity, supporting the idea of this as the primary site of viral replication in ferrets (Au et al., 2022; Ciurkiewicz et al., 2022). Meanwhile, in humans, SARS-CoV-2 replicates in the upper respiratory tract (nose, throat, and sinuses) as well as the lower respiratory tract (lungs, and airways) (V'Kovski et al., 2021). Infectious virus can be isolated from nasal samples 3-7 days post infection (dpi) onwards, and upper respiratory tract viral loads peak between 4 and 7 dpi (Everett et al., 2021; Au et al., 2022). Importantly, neither infectious virus nor viral antigen has been observed in the lungs (Au et al., 2022; Everett et al., 2021; Ciurkiewicz et al., 2022). This highlights the utility of ferrets for upper respiratory tract infection studies but limits their usefulness as a model of human lower respiratory tract infection. Notably, some strains of SARS-CoV-2 (such as the VOC omicron) have been shown to produce greater clinical manifestations within the upper respiratory tract, for which ferrets may be a useful model (Nori and Ghani Zghair, 2022).

Compared with small-animal models, NHPs present unique differences that can make them particularly useful as they offer the closest genetic, physiological, anatomical and developmental similarities to humans (Vallender and Miller, 2013). Several NHP species, including rhesus macaques, cynomolgus macaques and African green monkeys, have been utilized in previous research with related coronaviruses SARS-CoV and Middle East respiratory syndrome (MERS) (Rowe et al., 2004; Lawler et al., 2006; Yao et al., 2014; Kuiken et al., 2003; Qin et al., 2005; McAuliffe et al., 2004; de Wit et al., 2013).

Following SARS-CoV-2 challenge, rhesus macaques showed minimal clinical presentation, with some studies reporting mildly elevated scores while others noted no clinical signs of disease (Choudhary et al., 2022; Zheng et al., 2020; Shan et al., 2020; Speranza et al., 2022). Of those animals that did show clinical manifestations of disease, the latter returned to baseline within two weeks of challenge. However, viral RNA was detectable in the lungs and by nasopharyngeal swabs (Choudhary et al., 2022; Zheng et al., 2020; Shan et al., 2020; Speranza et al., 2022). Pneumonia presentation was variable, generally noted in the inferior lobes, with edema, hemorrhage, necrosis, alveolar wall thickening, endothelial damage, fibrosis and immune cell infiltration (Choudhary et al., 2022; Shan et al., 2020; Speranza et al., 2022). There was also evidence of myocarditis (Box 2), microthrombi, increased coagulation, cardiomyocyte disarray and necrosis following viral challenge (Feng et al., 2021; Rabbani et al., 2022). As with humans, these macaques showed age-related differences in disease presentation, with older animals exhibiting higher clinical scores, slower recovery, increased pulmonary infiltrates, and elevated levels of various cytokines (Speranza et al., 2022).

Studies using cynomolgus macaques showed similar results, with minimal clinical presentations in young, previously healthy animals (Urano et al., 2021; Goncalves et al., 2021; Salguero et al., 2021; Boszormenyi et al., 2021). In one study, pneumonia was confirmed in all animals by computed tomography despite the lack of clinical signs (Urano et al., 2021). In another, it was noted that, while there was no gross pathology, histologically there were changes in all animals (Salguero et al., 2021). These changes included alveolar necrosis, thickened alveolar walls, degeneration and sloughing of bronchiolar epithelium, and hyperemia in the caudal pulmonary lobes (Urano et al., 2021; Salguero et al., 2021; Boszormenyi et al., 2021). As with rhesus macaques, there were age-related changes, with elderly animals (23-30 years) having elevated clinical scores, longer periods of viral RNA detection, longer periods of pneumonia (including recurrence) and more-diffuse viral detection (including bronchi, lymph nodes, heart, liver and kidney) (Urano et al., 2021). Overall, rhesus and cynomolgus macaques exhibited similar infection kinetics, effects on organ systems and age-related differences of disease presentation that model that of mild COVID-19 disease in humans.

Although less common, African green monkeys (AGMs) have also been assessed as a potential SARS-CoV-2 model. Similar to both macaque species, AGMs showed mild clinical disease in young, previously healthy animals (Woolsey et al., 2021; Blair et al., 2021; Cross et al., 2020; Hartman et al., 2020). During the acute stage of infection, there were signs of systemic inflammation, with a transient decrease in lymphocytes and platelets, and an increase in neutrophils (Table 1) (Hartman et al., 2020; Woolsey et al., 2021). Histological investigations showed lung damage (detailed in Table 1) (Woolsey et al., 2021; Cross et al., 2020; Hartman et al., 2020). Age-related differences included development of ARDS leading to fatality (Blair et al., 2021). Lethal cases showed respiratory distress and evidence of systemic inflammatory response syndrome (SIRS) (Table 1) (Blair et al., 2021).

Overall, these three NHP models offer valuable insights into COVID-19 pathogenesis, immune responses and age-related differences. They closely resemble human disease presentation and are particularly useful for studying severe cases, cytokine profiles, cardiac involvement and vascular aspects of the disease. However, use of NHPs in research can be challenging due to the high cost of care, long breeding cycles, long life span and ethical concerns.

## Neurological disease

### Human disease manifestations

The prevalence of neurological symptoms associated with SARS-CoV-2 infection varies widely among reports, ranging from 36-82% (Ellul et al., 2020; Mao et al., 2020a; Liotta et al., 2020). Common neurological symptoms associated with SARS-CoV-2 infection include anosmia (loss of smell), dysgeusia (loss of taste), fatigue, myalgia (muscle pain) and headache (García-Azorín et al., 2020; Liotta et al., 2020; Merkler et al., 2020; Varatharaj et al., 2020; Yaghi et al., 2020; Lou et al., 2021; Oosthuizen et al., 2021). Various factors, including age, sex, pre-existing neurological disorders, comorbidities and the severity of COVID-19, influence the likelihood of experiencing neurological symptoms (García-Azorín et al., 2020; Liotta et al., 2020; Mao et al., 2020a).

The pathophysiology of neurological symptoms in COVID-19 remains complex and only partly understood. Autopsy studies have provided some insights into potential mechanisms (von Weyhern et al., 2020; Solomon et al., 2020). Lesions associated with acute hypoxia were common in the cerebrum and cerebellum, but it was noted that these lesions might be normal findings within an autopsied brain or a result of systemic acute hypoxia due to severe pulmonary disease (von Weyhern et al., 2020; Solomon et al., 2020). One study found only evidence of hypoxic changes (Solomon et al., 2020), while another observed encephalitis (Box 2), meningitis and neuronal cell death (von Weyhern et al., 2020). While there are conflicting reports as to whether SARS-CoV-2 is neuroinvasive, immunohistochemistry of the viral spike glycoprotein has shown positive results in cortical neurons (Song et al., 2021). The variability in neuropathological findings suggests that multiple factors, including host response and disease severity, contribute to the diverse neurological manifestations seen in COVID-19 patients.

### Rodent models

Expression of ACE2 protein in neuronal cells is thought to be necessary for viral neuroinvasion, although secondary mechanisms of systemic spread, such as extracellular vesicle formation could also contribute (Tahyra et al., 2022; Wang et al., 2020). Blocking the ACE2 receptor in human brain organoids resulted in a significant inhibition of SARS-CoV-2 infection (Song et al., 2021; Fumagalli et al., 2022). This might explain why CNS infection in humans is relatively rare, but relatively common in transgenic mouse models that overexpress hACE2 (McCray et al., 2007; Dinnon et al., 2020; Kumari et al., 2021). Due to the artificial K18 or Hfh4-driven overexpression, the expression pattern of hACE2 in these mice does not mimic that of natural expression in humans; therefore, results obtained from studies involving these animal models should be interpreted with caution (Dinnon et al., 2020; Renn et al., 2021; Amruta et al., 2023). Although it is likely that the mechanism of fatal encephalitis is similar between these animal models, here we will discuss only the K18-hACE2 mouse, as there is a relative paucity of information about the Hfh4-hACE2 model (Dinnon et al., 2020; Kumari et al., 2021).

Following intranasal SARS-CoV-2 inoculation, a high proportion of K18-hACE2 mice develop dose-dependent, fatal, non-suppurative (Box 2) meningoencephalitis (Vidal et al., 2022; Fumagalli et al., 2022; Zheng et al., 2021). However, studies have shown that these mice also develop anosmia and other neurological symptoms, including seizures, tremors, proprioceptive defects and abnormal gait, which underscores their continued potential as a model for neurological COVID-19 (Ye et al., 2021; Zheng et al., 2021; Fumagalli et al., 2022; Kumari et al., 2021; Carossino et al., 2022).

Infection of K18-hACE2 mice was associated with high titers of viral RNA and infectious viral particles in the CNS (Fumagalli et al., 2022; Vidal et al., 2022). Histological examinations showed diffuse positive staining for SARS-CoV-2 proteins throughout the cerebrum, with limited immunopositivity in the cerebellum (Song et al., 2021; Fumagalli et al., 2022). Neurons themselves appeared to be the primary target, while astrocytes (Box 2) and microglia (Box 2) were associated with viral positivity in some but not all studies (Fumagalli et al., 2022; Song et al., 2021; Golden et al., 2020; Carossino et al., 2022). Evidence suggested that intracerebral spread occurred via neuronal synapses (Box 2) (Vidal et al., 2022). While epithelial cells were susceptible to viral infection, no viral protein was observed in the choroid plexus (Box 2) epithelium, suggesting that neuroinvasion was not occurring via the cerebrospinal fluid (CSF) (Ng et al., 2023; Vidal et al., 2022; Song et al., 2021).

SARS-CoV-2 encephalitis in K18-hACE2 mice was characterized by perivascular cuffing (Box 2), neuronal necrosis (Letsinger et al., 2023) and immune cell infiltration of the brain (Fumagalli et al., 2022; Vidal et al., 2022). Furthermore, many studies found evidence of activated microglial cells and astrocytes (Fumagalli et al., 2022; Vidal et al., 2022; Letsinger et al., 2023). Cerebral vasculitis (Box 2) and the resulting fibrin thrombi were associated with significant disruption and remodeling of the brain vasculature (Vidal et al., 2022; Letsinger et al., 2023; Song et al., 2021), which might model the hypoxic lesions observed in human COVID-19 patients; however, this might be a weak association as no ischemic necrosis was noted (Vidal et al., 2022). Analogous to human patients, viral infection of mouse neurons was associated with vacuolar degeneration (Box 2), neuronal necrosis, satellitosis (Box 2) and neurophagia (Box 2) (Vidal et al., 2022; Letsinger et al., 2023). Altogether, the current evidence suggests that K18-hACE2 mice acquire diffuse brain lesions with high viral loads, limiting their translatability.

The neuroinvasive mechanism of SARS-CoV-2 in K18-hACE2 mice remains unclear and might depend on the route of infection (Bleau et al., 2015; Li et al., 2020; Bilinska et al., 2021; Meinhardt et al., 2021; Patrì et al., 2021; Rhea et al., 2021; Bauer et al., 2022; Fumagalli et al., 2022; Fontes-Dantas et al., 2023). Viral infection of olfactory epithelium has been observed to progress to neuronal necrosis in the olfactory bulb, leading to diffuse viral encephalitis. This suggests that retrograde viral migration in olfactory nerve axons is the route of viral neuroinvasion (Ye et al., 2021; Vidal et al., 2022; Letsinger et al., 2023). Notably, viral encephalitis was circumvented in K18-hACE2 mice inoculated via aerosolized virus (Fumagalli et al., 2022). Additionally, viral protein, viral RNA, infectious particles and infection-associated lesions were not detected in the brain of aerosol challenged mice (Fumagalli et al., 2022). The absence of viral RNA in the serum of both aerosol- and intranasal-challenged mice (Winkler et al., 2020; Kumari et al., 2021; Zheng et al., 2021; Fumagalli et al., 2022) suggests that viral translocation across the blood−brain barrier (BBB) does not play a major role in neuroinvasion in the K18-hACE2 mouse, although the possibility cannot be completely excluded (Li et al., 2020; Meinhardt et al., 2021; Vidal et al., 2022; Fumagalli et al., 2022). Moreover, the differences in neuroinvasion between intranasal inoculation and aerosolization might reflect a bolus or dose phenomenon, with aerosolization representing a more natural and translatable route of infection and subsequent disease progression.

In multiple different inbred strains of mice, intranasal challenge of SARS-CoV-2 MA10 led to a decrease in markers of BBB integrity (Amruta et al., 2023). Specifically, in BALB/c mice, there was an increase in microglial cell activation within cortical regions compared to those in unchallenged control mice (Amruta et al., 2023). BALB/c mice also had increased perivascular cuffing; however, fatal encephalitis did not develop and viral proteins were not detected in the brain of any observed mice (Amruta et al., 2023). MA10 was capable of inducing neuroinflammation and neuropathogenesis without causing diffuse CNS infection (Amruta et al., 2023), making it a more suitable model for acute neuro-COVID-19 compared to K18-hACE2 and Hfh4-hACE2 mice. Additionally, MA10 infection in mice deficient in caveolin-1, a protein essential for BBB permeability, was found to result in increased BBB permeability, disruption of BBB tight junctions, and deficits in learning and memory, highlighting the versatility of MA10 for use in mice of different genetic backgrounds (Trevino et al., 2024).

Other mouse models have been developed by using exposure to different viral variants and even components of the virus itself. For instance, infusion of SARS-CoV-2 spike protein into the brains of outbred mice induced late cognitive impairment, dysfunction and neuroinflammation (Fontes-Dantas et al., 2023). Additionally, infection with the SARS-CoV-2 Beta variant in C57BL/6 mice led to CNS infiltration of monocytes and microglial cell activation (Vanderheiden et al., 2024).

In hamsters, SARS-CoV-2 primarily manifested as a respiratory disease, with limited reports of neurological symptoms (Luan et al., 2020; Monchatre-Leroy et al., 2021; Käufer et al., 2022). However, some studies noted transient anosmia and ageusia − akin to clinical human symptoms − in infected hamsters (Xydakis et al., 2020; Golden et al., 2022; Carpenter et al., 2023; Bryche et al., 2020; de Melo et al., 2021; Käufer et al., 2022). This might be due to neurological deficits, or to damage to the olfactory epithelium in the nasal turbinates and associated loss of cilia necessary for odor detection (de Melo et al., 2021). While no viral protein was found in Syrian hamster brains by immunohistochemistry, isolated brain homogenates at day 3 post challenge showed infectious viral particles that had cleared by day 6, suggesting a transient and non-productive CNS infection (Bryche et al., 2020; Monchatre-Leroy et al., 2021; Käufer et al., 2022). Viral RNA was found in various brain regions, including the olfactory bulb, brainstem, cerebral cortex and cerebellum, as well as in the serum (Monchatre-Leroy et al., 2021; de Melo et al., 2021). Notably, one study observed early accumulation of proteins associated with neurodegenerative disorders in cortical neurons, but no evident neurodegeneration (Käufer et al., 2022). This was associated with microgliosis in the olfactory bulb following viral clearance, indicating ongoing neuroinflammatory processes. However, viral antigens have not been found in the brain, suggesting that diffuse infection CNS and COVID-19 disease does not occur in this hamster model (Bryche et al., 2020; de Melo et al., 2021).

Roborovski dwarf hamsters seemed similarly resistant to CNS infection (Trimpert et al., 2020). No viral RNA signals were detected in the brain of infected hamsters but blood leukocytes observed in cerebral vessels suggested that, in this model, viral dissemination to the brain may occur through the vasculature (Trimpert et al., 2020). Hamster cerebellar organotypic slice cultures were used to examine SARS-CoV-2 infection in the absence of the BBB, and viral replication was limited to 1 dpi, with a subsequent decline in viral RNA (Lamoureux et al., 2022). Likewise, there was no evidence of cell-to-cell transmission of the virus or neuronal infection, unlike what was observed during human autopsies (Stein et al., 2022).

In contrast, transgenic K18-hACE2 hamsters developed severe lesions in both nasal cavity and CNS in response to infection with SARS-CoV-2 (Golden et al., 2022). The nasal cavity showed extensive damage and inflammation of the olfactory epithelium; widespread brain disease included multifocal gliosis, meningitis, perivascular cuffing and neuronal necrosis (Golden et al., 2022). Viral RNA was detected in the olfactory epithelium, cerebral cortex and cerebellum at day 5 post challenge and persisted at a reduced level until 15 dpi (Golden et al., 2022). The presence of viral antigen in neurons and microglial cells confirmed true CNS infection in this model (Golden et al., 2022). These findings suggest that K18-hACE2 hamsters, like K18-hACE2 mice, are highly susceptible to CNS-lesion-associated disease (Golden et al., 2022). Overall, since widespread expression of human ACE2 is likely to make it susceptible to viral infection of the CNS, the K18-hACE2 hamster might be a useful model to further assess certain aspects of neurological COVID-19, such as anosmia and ageusia as well as mechanisms of viral neuroinvasion.

### Non-rodent models

Ferrets have been shown to comprise viral RNA present within various brain regions, including the olfactory bulb, cerebrum and cerebellum following both intranasal and intratracheal challenge, although neuroinvasive processes appear to be more efficient following intranasal challenge (van de Ven et al., 2021). This suggests viral dissemination via the circulatory system and subsequent brain seeding, which is in alignment to some human studies (Jacobs et al., 2022; Stein et al., 2022). In some ferret studies, viral RNA in the brain was detected as early as day 3 and until 7 dpi (Schlottau et al., 2020; Everett et al., 2021; Au et al., 2022; Ciurkiewicz et al., 2022). Despite this, there have been no reports of ferrets displaying any evidence of SARS-CoV-2-associated neurological signs or neuropathy (Schlottau et al., 2020; Everett et al., 2021; Monchatre-Leroy et al., 2021; van de Ven et al., 2021; Au et al., 2022; Ciurkiewicz et al., 2022). While ferrets may provide insight into the neuroinvasive mechanisms of SARS-CoV-2 and viral transmission, they do not appear to be a suitable model for the neurological disease manifestations of COVID-19 (Kutter et al., 2021; Kim et al., 2020; Richard et al., 2020).

In the brain of NHPs, significant lesions have been observed post viral infection (Choudhary et al., 2022; Philippens et al., 2022; Rutkai et al., 2022). Multiple inoculation methods, including standard intranasal and aerosol methods as well as intracranial exposure, have been used (Jiao et al., 2021; Beckman et al., 2022; Choudhary et al., 2022; Philippens et al., 2022; Rutkai et al., 2022); however, to date, no significant differences in brain pathology have been noted based on route of infection (Rutkai et al., 2022).

Neural pathology has primarily been observed in rhesus macaques (Rutkai et al., 2022); however lesions have also been observed in cynomolgus macaques (Philippens et al., 2022) and African green monkeys (Rutkai et al., 2022). One study found that basal ganglia, brainstem and cerebellum exhibit most significant injury and pathology among infected animals (Rutkai et al., 2022). Observations of multifocal acute microhemorrhages, primarily within these regions, were common but not always associated with ischemic injury, and often lacked evidence of thrombi or vascular injury (Rutkai et al., 2022). Neuronal changes were most often found in the cerebellum and brainstem, with marked neuronal and surrounding cell injuries, possibly due to hypoxia in these brain regions, as indicated by markers in the vasculature of the brain stem and basal ganglia (Rutkai et al., 2022). Neuronal cell death was also noted in the entorhinal cortex (Box 2), hippocampus, thalamus and midbrain (Jiao et al., 2021). Evidence of microglial activation was observed in various brain regions, including the pituitary gland, olfactory bulb and cerebellum (Philippens et al., 2022). In aged rhesus macaques, activated microglial cells were associated with neuronal synaptic engulfment and myelin degradation, indicating a potential link between SARS-CoV-2-associated neuroinflammation and neurodegenerative processes (Beckman et al., 2022). There was also evidence of astrocyte activation (Rutkai et al., 2022; Beckman et al., 2022), glial cell hyperplasia (Jiao et al., 2021) and elevated brain cytokines, indicating further neuroinflammatory processes (Jiao et al., 2021).

There is limited evidence for vascular dissemination being the pathway of neuroinvasion in NHPs; however, some studies observed perivascular cuffing without significant parenchymal lymphocyte infiltration (Choudhary et al., 2022; Rutkai et al., 2022), while another study found evidence of T-cell infiltration of the pituitary gland and multiple parts of the brain parenchyma ∼35 dpi (Philippens et al., 2022). This suggests that translocation of immune cells from the peripheral blood supply to the brain is possible in response to infection (Philippens et al., 2022), but whether the virus can move similarly has not been shown. In contrast, two studies support retrograde axonal transport via the olfactory nerve as the primary mechanism of neuroinvasion (Jiao et al., 2021; Beckman et al., 2022). In a study of rhesus macaques, intranasal infection led to detection of viral RNA and antigen in the nasal mucosa, olfactory tract and CSF as early as 1 dpi (Jiao et al., 2021). Viral RNA and antigen were observed migrating into the olfactory trigone (Box 2) by 4 dpi and into the entorhinal cortex by 7 dpi (Jiao et al., 2021).

While there is evidence for neuroinvasion in NHPs, viral replication may not be well supported (Jiao et al., 2021). Detection of viral protein in NHP brains largely appears to be limited to vascular cells, but some studies have found viral proteins in areas of inflammation associated with neurons, glial cells and immune cells (Rutkai et al., 2022; Philippens et al., 2022; Choudhary et al., 2022; Beckman et al., 2022). These differences may have been due to the age of the animals in the study, as older rhesus macaques displayed a higher level of viral burden in neurons, astrocytes and microglial cells when compared to their younger counterparts (Beckman et al., 2022). Analyses of viral RNA in the CNS were variable, with some studies finding scant viral RNA in a small number of animals in multiple regions in the brain (Rutkai et al., 2022; Philippens et al., 2022), while others found none (Choudhary et al., 2022; Jiao et al., 2021). None of these studies observed cerebral sub-genomic RNA, which is indicative of viral replication (Jiao et al., 2021; Choudhary et al., 2022; Philippens et al., 2022; Rutkai et al., 2022). Altogether, NHPs have exhibited neuropathology associated with SARS-CoV-2 infection that make them suitable models for acute neurological COVID-19 studies.

## GI disease

### Human disease manifestations

Gastrointestinal (GI) symptoms, including symptoms of diarrhea, nausea or vomiting, loss of appetite and abdominal pain have been observed in a subset of patients (de Oliveira et al., 2020; Jin et al., 2020; Mao et al., 2020b; Pan et al., 2020; Sultan et al., 2020; Carnevale et al., 2021; Din et al., 2021). Several mechanisms have been proposed to explain GI involvement in COVID-19, including direct viral infection of GI epithelium, secondary damage due to a cytokine storm and gut microbial dysbiosis (Lamers et al., 2020; Lehmann et al., 2021; Serban et al., 2021; Yeoh et al., 2021; Zuo et al., 2020).

Viral RNA detected in patient fecal samples suggests that the intestine may be an extra-pulmonary site of SARS-CoV-2 replication and that fecal-oral transmission may be possible (Chen et al., 2020; Zuo et al., 2020). Small-intestinal epithelial cells express the ACE2 receptor and are susceptible to viral replication (Shang et al., 2020; Xiao et al., 2020; Zhang et al., 2020; Patankar et al., 2021). The disruption of normal cellular function or death of enterocytes due to viral infection is thought to be one of the causes of GI symptoms observed in some patients (Lehmann et al., 2021). Additionally, a cytokine-storm-induced prothrombotic state can also lead to microthrombi and ischemic infarcts in multiple body systems, including the GI tract (Savla et al., 2021; Serban et al., 2021; Wu et al., 2022). Mesenteric thrombosis and ischemia are rare COVID-19 complications that can significantly increase morbidity and mortality (Serban et al., 2021; Wu et al., 2022).

**Fig. 3.**
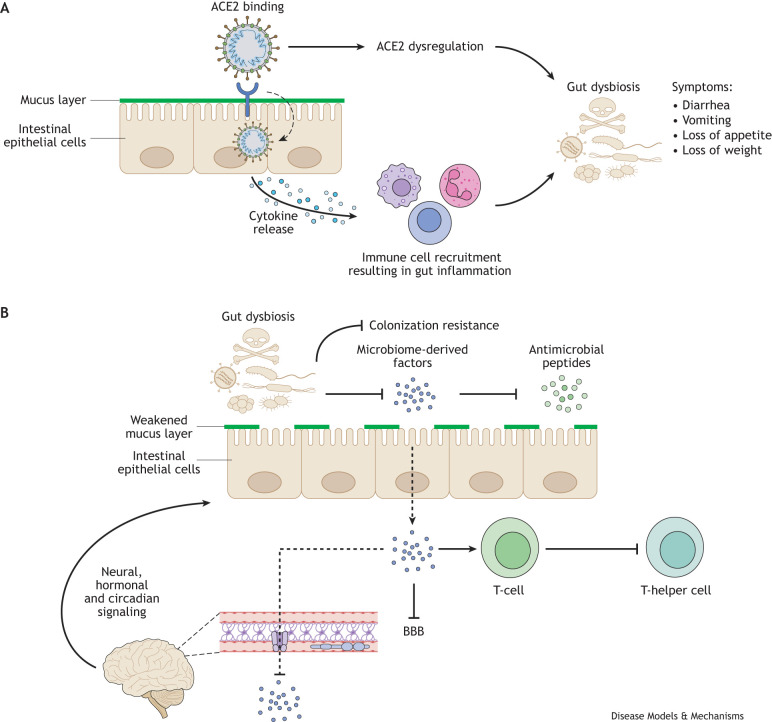
**The effects of SARS-CoV-2 infection on the gut–brain axis.** Two proposed mechanisms for SARS-CoV-2-associated gut dysbiosis include (A) ACE2 dysregulation and gut enteric inflammation arising from viral infection of enterocytes. Gut dysbiosis is associated with clinical signs including diarrhea, vomiting, loss of appetite and weight. (B) The bidirectional communication that occurs between the enteric nervous system and the central nervous system is influenced by the gut microbiota. Factors produced by commensal gut microbes can traverse from the epithelium and through the blood-brain barrier (BBB; dashed lines) and impact BBB integrity (Braniste et al., 2014), the development of neurons (Yang et al., 2020) and glial cells (Erny et al., 2015), and inflammatory signaling associated with those cells (Soliman et al., 2013). Neural, endocrine and circadian signals from the brain influence mucus and biofilm production, motility, intestinal permeability and immune function in the gut, which in turn, influences gut microbiota composition. The gut microbiota also play a major role in the health and maintenance of the body through its influences on local and systemic immunity. The presence of commensal gut microbiota provides colonization resistance, preventing the proliferation of pathogenic or invading microbiota (Khan et al., 2021). Microbiome-derived factors influence mucus production (Willemsen et al., 2003), the production of anti-microbial peptides (Kinnebrew et al., 2010) and the differentiation of T-cells into T-helper cells or T-cells with a regulatory phenotype (Atarashi et al., 2011; Mazmanian et al., 2005). Blunt arrows indicate processes that are inhibited when the normal commensal microbiota population is disrupted. Therefore, SARS-CoV-2-associated gut dysbiosis can negatively affect local gut and systemic immune responses, resulting in increased disease at both local and distal sites.

SARS-CoV-2 has been shown to significantly alter the normal gut microbiome, resulting in a loss of bacterial richness and diversity (Gu et al., 2020; Yeoh et al., 2021). This dysbiosis increases the likelihood of illness from opportunistic pathogens, and has downstream effects on the immune system and the CNS (Fig. 3) (Li et al., 2019; Zuo et al., 2020; Yeoh et al., 2021; Wiertsema et al., 2021). The bidirectional interaction that occurs between the gut and the CNS, often referred to as the gut−brain axis, is primarily mediated via gut microbes, and the hormonal, endocrine and circadian signals relayed to the gut from the brain (Carabotti et al., 2015; Silva et al., 2020; Parker et al., 2020). Therefore, any disruption to normal gut microbiota can have downstream effects on the CNS (Fig. 3B). In addition, dysbiosis increases patient susceptibility to sepsis, hospital-acquired infections and organ failure, and has been associated with severe illness in hospitalized patients (McDonald et al., 2016).

One possible proposed mechanism of SARS-CoV-2-induced gut dysbiosis is virus-associated intestinal inflammation; however, it is still unclear whether dysbiosis leads to inflammation, inflammation leads to dysbiosis, or if there exists a cyclical relationship between the two (Clerbaux et al., 2022). Another proposed mechanism involves the dysregulation of ACE2 due to SARS-CoV-2 binding in the gut (Clerbaux et al., 2022). Regardless of mechanism, a loss of commensal symbionts known to have immunomodulatory effects, such as *Faecalibacterium prausnitzii*, *Eubacterium rectale* and *Bifidobacteria*, as well as enrichment of opportunistic pathogens was observed in human patients. This dysbiosis persisted even after patients had been medically cleared and recovered from COVID (Zuo et al., 2020). This type of dysbiosis is associated in other diseases with increased severity, suggesting that the gut microbiome plays a role by modulating the host immune response (Yeoh et al., 2021).

### Rodent models

Few mouse studies have assessed the effects of SARS-CoV-2 infection on the gastrointestinal tract. One study that looked at gut microbiome diversity and richness during infection in the K18-hACE2 mouse model observed that high-dose inoculation led to greater disruption of microbial diversity and richness in the cecum, reduced relative abundance of Firmicutes and Actinobacter, and a reduced Firmicutes:Bacteroidetes ratio compared to control mice (Seibert et al., 2021). The increased abundance of families within the Firmicutes phylum was positively correlated with severity of viral infection in the lung (Seibert et al., 2021).

Syrian hamsters − although no overt clinical symptoms, such as diarrhea − had been observed, were also used to study SARS-COV-2-associated dysbiosis (Seibert et al., 2022; Sencio et al., 2022). Non-infectious viral RNA was found in the colon at 2 dpi, and little to no viral RNA was found in the small intestine and cecum (Sencio et al., 2022). Cytokines in the colon were upregulated, but no evidence of intestinal damage was observed (Sencio et al., 2022). Some evidence of intestinal barrier dysfunction was observed, with elevated intestinal fatty-acid-binding protein in the blood (Sencio et al., 2022). As with the mouse study and in human patients, changes in the gut microbiome composition were noted by 16S ribosomal RNA sequencing (Sencio et al., 2022; Seibert et al., 2022). On principal coordinate analysis, there was a clustering of gut microbiome composition across time, suggestive of a significant shift following SARS-CoV-2 infection (Seibert et al., 2022; Sencio et al., 2022), which correlated with disease severity and inflammation (Sencio et al., 2022). There was also an enrichment of opportunistic pathogens that positively correlated with viral RNA and pathology in the cecum, suggesting that microbiome changes may secondarily modulate disease severity in the gut (Seibert et al., 2022).

### Non-rodent models

While studies into gastrointestinal manifestations of COVID-19 have not been performed in ferrets, rhesus macaques and cynomolgus macaques have been used to study SARS-CoV-2-associated gut dysbiosis (Sokol et al., 2021). While neither weight loss nor overt signs of pneumonia were noted, occasional and transient diarrhea was observed (Sokol et al., 2021). Viral RNA was detected in the rectal fluid of one cynomolgus and one rhesus macaque at 7 dpi (Sokol et al., 2021). Notably, one of these monkeys still had RNA-positive rectal samples at 26 dpi, suggesting that SARS-CoV-2 can persist in or shed through the gastrointestinal tract (Sokol et al., 2021). There were alterations in the fecal microbiota composition following infection with this disruption in the microbial ecosystem peaking at 10−13 dpi (Sokol et al., 2021). The relative abundance of *Streptococcus* species was strongly and positively correlated with select plasma chemokine levels, strengthening the link between gut dysbiosis and immune response (Sokol et al., 2021). Altogether, NHPs infected with SARS-CoV-2 showed evidence of gut microbiota disturbances and occasional diarrhea, with changes in gut microbiota composition associated with immune responses.

Considering the impact of altered gut microbiota composition on COVID-19 disease severity, it is important to conduct additional investigations that aim to detail the molecular mechanisms through which SARS-CoV-2 infections alter the gut microbiome and how these changes influence inflammation and COVID-19 severity. Furthermore, modulating the gut microbiota composition for potential mitigation of disease severity might prove to be an additional tool in disease management in a clinical setting. Animal models will be critical in testing hypotheses pertaining to the interaction between gut microbiota and COVID-19 severity.

## PASC

### Human disease manifestations

Post-acute sequelae of COVID-19 (PASC), also referred to as long COVID, represents a significant concern in the context of SARS-CoV-2 infection. It encompasses the persistence or emergence of new clinical manifestations for 30 or more days following infection (Thaweethai et al., 2023). High-risk factors for PASC include being female, a member of an ethnic minority group, of low socioeconomic status, obese and having other comorbidities (Subramanian et al., 2022). Severe or mild acute COVID-19 illness can lead to PASC symptoms, suggesting that factors beyond viral infection, such as stress or other comorbidities, can contribute to the presentation of PASC symptoms (Hernandez-Romieu et al., 2022; Augustin et al., 2021). PASC prevalence is lower in those infected with the Omicron variant, previously vaccinated or treated with antiviral therapy, indicating that these factors influence its occurrence (Sherif et al., 2023).

Common symptoms associated with PASC include fatigue, post-exertional malaise, cognitive difficulties (often described as brain fog) and joint pain (Thaweethai et al., 2023). Fatigue and post-exertional malaise stand out as the most frequently reported and debilitating symptoms (Thaweethai et al., 2023). PASC prevalence estimates vary across different regions and populations, but it has been estimated that 6% of the adult population in the USA [see National Center for Health Statistics (CDC): Long COVID Household Pulse Survey (https://www.cdc.gov/nchs/covid19/pulse/long-covid.htm, accessed 3 September 2024], 2.9% of the entire population in the UK [see Office for National Statistics (ONS): Prevalence of ongoing symptoms following coronavirus (COVID-19) infection in the UK: 30 March 2023 (https://www.ons.gov.uk/peoplepopulationandcommunity/healthandsocialcare/conditionsanddiseases/bulletins/prevalenceofongoingsymptomsfollowingcoronaviruscovid19infectionintheuk/30march2023)] and 8.9% of 20,000 patients surveyed in Shanghai, China, reported experiencing PASC symptoms (Cai et al., 2023) might be experiencing PASC. To the global economy PASC is estimated to contribute to a loss of ∼1 tn US dollars every year (Al-Aly et al., 2024). Further investigation into the mechanisms of disease is crucial due to the detrimental impact of PASC on both clinical health and the economy, highlighting the need for additional research to identify effective treatments and preventatives.

The mechanisms underlying PASC are complex, probably multifactorial. They might include immune dysregulation; microbiota dysbiosis; autoimmunity and priming; blood clotting and endothelial abnormalities; dysfunctional neurological signaling; reactivation of other viruses, such as the Epstein-Barr virus; and viral persistence (de Melo et al., 2021; Natarajan et al., 2022; Stein et al., 2022; Craddock et al., 2023). However, the exact mechanisms underlying the syndrome are unclear (Calabrese et al., 2021; Pretorius et al., 2021; Liu et al., 2022b; Peluso and Deeks, 2022; Phetsouphanh et al., 2022; Seeßle et al., 2022; Su et al., 2022). Evidence indicates that SARS-CoV-2 RNA or antigen can persist in humans for extended periods of time (Stein et al., 2022; Appelman et al., 2024; Proal et al., 2023). PCR analysis of tissues obtained from autopsies of 44 unvaccinated individuals who had died from COVID-19 between 26 April 2020 and 2 March 2021 revealed viral RNA in various parts of the body − including the brain, respiratory system, cardiovascular system and gastrointestinal system − up to 230 days after the initial infection (Stein et al., 2022). In one patient, subgenomic RNA − which is generated by the virus during active replication and signifies ongoing viral replication − was also detected in the lungs as late as 76 dpi (Stein et al., 2022). Despite these findings, no infectious virus was isolated from any samples obtained from the more chronically infected patients (Stein et al., 2022). Additionally, the presence of viral RNA in patient fecal samples up to 210 dpi suggests that the GI tract may be a viral reservoir (Natarajan et al., 2022). Analysis of patient blood samples revealed the presence of viral proteins but not RNA, suggesting that viral persistence occurs primarily within tissues (Proal et al., 2023). Altogether, the data suggest that viral persistence can be observed in multiple tissue types; however, the connection between viral persistence and PASC symptoms remains to be determined.

Significantly more IFN-gamma- and TNF-alpha-producing SARS-CoV-2-specific T-cells were found in the blood of patients who got infected with SARS-CoV-2 and had been diagnosed with PASC compared to those who recovered from COVID-19 without PASC (Littlefield et al., 2022). The increased frequency of these T-cells was also associated with increased systemic inflammation and decreased lung function, suggesting that these T-cells contribute to symptoms of pulmonary PASC, and the increased frequency of SARS-CoV-2-specific T-cells was attributed to a viral reservoir (Littlefield et al., 2022). Additionally, analysis of blood obtained from patients diagnosed with PASC or not at 8 months post infection with SARS-CoV-2 revealed markers of T-cell exhaustion in those diagnosed with long COVID (Yin et al., 2024). T-cell exhaustion suggests that ongoing viral stimulation occurs in patients with PASC symptoms (Yin et al., 2024). The same study found more SARS-CoV-2 antibodies in patients diagnosed with long COVID than in recovered patients, further supporting the idea of a viral reservoir and immune disturbances caused by persistent virus presence (Yin et al., 2024). However, in patients who had previously experienced COVID-19, those experiencing the PASC symptom of post-exertional malaise and those who had recovered, both had viral N protein within their skeletal muscle tissue (Appelman et al., 2024). There were no differences in the level of N protein between PASC and recovered patients, suggesting that factors other than viral persistence were playing a role in the pathophysiology of post-exertional malaise in PASC patients, highlighting the need to further explore mechanisms of PASC pathophysiology (Appelman et al., 2024).

### Potential animal models

The current lack of established animal models for PASC underlines the challenges associated with performing PASC studies using animals (Frere et al., 2022; Jansen et al., 2022). Overcoming those challenges could expedite the development of PASC models.

Efforts are underway to develop and validate a PASC animal model (Jansen et al., 2022; Usai et al., 2023). At 60 dpi BALB/c mice infected with SARS-CoV-2 MA10 showed signs of neuroinflammation and neuropathogenesis, suggesting this model to be suitable to investigate the long-term neuropathological changes associated with SARS-CoV-2 infection (Gressett et al., 2023 preprint). Such neurological insults could have enduring impacts on behavior and cognitive function, which can be explored in future long-term studies using behavioral and physiological assays. MA10 infection in mice also resulted in chronic pulmonary damage and fibrosis, symptoms as observed in humans recovering from diffuse alveolar damage (Dinnon et al., 2022). Infected hamsters had chronic inflammation in the olfactory bulbs and olfactory epithelium that correlated with a decrease in compulsive and anxiety-like behavior (Frere et al., 2022). NHPs have been shown to get acute neuroinflammation, neuronal and glial cell injury, and hypoxic injury associated with SARS-CoV-2 infection (Rutkai et al., 2022). Although the use of NHPs for long-term COVID research poses significant challenges, they appear to be a suitable model for neurological PASC since they show evidence of early neurological lesions that may have lasting effects and contribute to PASC symptoms. Murine models appear to be promising candidates for GI-PASC research because their gut microbiome can be manipulated more easily compared to other species (Hart et al., 2018; Park and Im, 2020; Manca et al., 2020). Further efforts to establish preclinical animal models for PASC will be essential in streamlining the development of therapeutics and preventatives.

## Conclusion

Over the past 4 years, an exceptional amount of data investigating SARS-CoV-2 infection has highlighted how investment in animal models of infectious disease can be mobilized to understand the pathogenic processes and mechanisms involved in acute disease (Table 1). As the world population now turns to dealing with an ongoing endemic disease and its longer-term manifestations, it is important to re-examine the utility of current animal models. Understanding the mechanisms of residual disease in various biological systems remains a key challenge. While the acute disease processes most dramatically involve the immune and pulmonary systems, the subacute and post-acute effects on the cardiovascular, neurological and gastrointestinal systems are more challenging to study in animal models. This Review has highlighted animals that demonstrate efficacy as models for acute disease processes involving these organ systems; however, the suitability of these animals as models for post-acute disease processes remains unclear.

To understand the complexities of COVID-19 infection, new and continued innovation in both animal models and *in vitro* systems, such as organoid models, is essential. A significant obstacle in developing animal models for COVID-19 is the high cost and limited access to biocontainment research facilities. Creating animal and organ system models of COVID-19 that can be studied outside of this restrictive environment would enable more extensive research, thereby accelerating the validation and creation of effective models. Alternatives to animal models, such as human organoids, show potential use in the study of pathological processes associated with SARS-CoV-2 infection (Ng et al., 2023). The current primary limitation with organoids is that any organoid lesions associated with viral infection cannot be correlated with clinical symptoms. Organoids also provide limited integration of multiple organ systems in comparison with animal models. Therefore, animal models remain critical for understanding the interactions between organ systems and the immune system, and how these contribute to the clinical manifestations of COVID-19.

Information presented here aims to guide the design of upcoming studies that explore post-acute disease processes within the organ systems discussed, which will play a pivotal role in developing effective treatments and preventative strategies for disease associated with SARS-CoV-2 infection.

## References

[DMM052086C1] Adu-Amankwaah, J., Mprah, R., Adekunle, A. O., Ndzie Noah, M. L., Adzika, G. K., Machuki, J. O. and Sun, H. (2021). The cardiovascular aspect of COVID-19. *Ann. Med.* 53, 227-236. 10.1080/07853890.2020.1861644 33345626 PMC7784826

[DMM052086C2] Al-Aly, Z., Davis, H., McCorkell, L., Soares, L., Wulf-Hanson, S., Iwasaki, A. and Topol, E. J. (2024). Long COVID science, research and policy. *Nat. Med.* 30, 2148-2164. 10.1038/s41591-024-03173-6 39122965

[DMM052086C4] Alexander, M. R., Schoeder, C. T., Brown, J. A., Smart, C. D., Moth, C., Wikswo, J. P., Capra, J. A., Meiler, J., Chen, W. and Madhur, M. S. (2020). Predicting susceptibility to SARS-CoV-2 infection based on structural differences in ACE2 across species. *FASEB J.* 34, 15946-15960. 10.1096/fj.202001808R 33015868 PMC7675292

[DMM052086C5] Amruta, N., Ismael, S., Leist, S. R., Gressett, T. E., Srivastava, A., Dinnon, K. H., 3rd, Engler-Chiurazzi, E. B., Maness, N. J., Qin, X., Kolls, J. K. et al. (2023). Mouse adapted SARS-CoV-2 (MA10) viral infection induces neuroinflammation in standard laboratory mice. *Viruses* 15, 114. 10.3390/v15010114 PMC9863644 36680154

[DMM052086C6] Appelman, B., Charlton, B. T., Goulding, R. P., Kerkhoff, T. J., Breedveld, E. A., Noort, W., Offringa, C., Bloemers, F. W., Van Weeghel, M., Schomakers, B. V. et al. (2024). Muscle abnormalities worsen after post-exertional malaise in long COVID. *Nat. Commun.* 15, 17. 10.1038/s41467-023-44432-3 38177128 PMC10766651

[DMM052086C7] Arce, V. M. and Costoya, J. A. (2021). SARS-CoV-2 infection in K18-ACE2 transgenic mice replicates human pulmonary disease in COVID-19. *Cell. Mol. Immunol.* 18, 513-514. 10.1038/s41423-020-00616-1 33446889 PMC7808118

[DMM052086C8] Atarashi, K., Tanoue, T., Shima, T., Imaoka, A., Kuwahara, T., Momose, Y., Cheng, G., Yamasaki, S., Saito, T., Ohba, Y. et al. (2011). Induction of colonic regulatory T cells by indigenous Clostridium species. *Science* 331, 337-341. 10.1126/science.1198469 21205640 PMC3969237

[DMM052086C9] Au, G. G., Marsh, G. A., McAuley, A. J., Lowther, S., Trinidad, L., Edwards, S., Todd, S., Barr, J., Bruce, M. P., Poole, T. B. et al. (2022). Characterisation and natural progression of SARS-CoV-2 infection in ferrets. *Sci. Rep.* 12, 5680. 10.1038/s41598-022-08431-6 35383204 PMC8981194

[DMM052086C10] Augustin, M., Schommers, P., Stecher, M., Dewald, F., Gieselmann, L., Gruell, H., Horn, C., Vanshylla, K., Cristanziano, V. D., Osebold, L. et al. (2021). Post-COVID syndrome in non-hospitalised patients with COVID-19: a longitudinal prospective cohort study. *Lancet Reg. Health Eur.* 6, 100122. 10.1016/j.lanepe.2021.100122 34027514 PMC8129613

[DMM052086C11] Ayoubkhani, D., Khunti, K., Nafilyan, V., Maddox, T., Humberstone, B., Diamond, I. and Banerjee, A. (2021). Post-covid syndrome in individuals admitted to hospital with covid-19: retrospective cohort study. *BMJ* 372, n693. 10.1136/bmj.n693 33789877 PMC8010267

[DMM052086C12] Bao, L., Deng, W., Huang, B., Gao, H., Liu, J., Ren, L., Wei, Q., Yu, P., Xu, Y., Qi, F. et al. (2020). The pathogenicity of SARS-CoV-2 in hACE2 transgenic mice. *Nature* 583, 830-833. 10.1038/s41586-020-2312-y 32380511

[DMM052086C13] Bauer, L., Laksono, B. M., De Vrij, F. M. S., Kushner, S. A., Harschnitz, O. and Van Riel, D. (2022). The neuroinvasiveness, neurotropism, and neurovirulence of SARS-CoV-2. *Trends Neurosci.* 45, 358-368. 10.1016/j.tins.2022.02.006 35279295 PMC8890977

[DMM052086C14] Becker, K., Beythien, G., De Buhr, N., Stanelle-Bertram, S., Tuku, B., Kouassi, N. M., Beck, S., Zickler, M., Allnoch, L., Gabriel, G. et al. (2021). Vasculitis and neutrophil extracellular traps in lungs of golden syrian hamsters with SARS-CoV-2. *Front. Immunol.* 12, 640842. 10.3389/fimmu.2021.640842 33912167 PMC8072219

[DMM052086C15] Beckman, D., Bonillas, A., Diniz, G. B., Ott, S., Roh, J. W., Elizaldi, S. R., Schmidt, B. A., Sammak, R. L., Van Rompay, K. K. A., Iyer, S. S. et al. (2022). SARS-CoV-2 infects neurons and induces neuroinflammation in a non-human primate model of COVID-19. *Cell Rep.* 41, 111573. 10.1016/j.celrep.2022.111573 36288725 PMC9554328

[DMM052086C16] Belser, J. A., Katz, J. M. and Tumpey, T. M. (2011). The ferret as a model organism to study influenza A virus infection. *Dis. Model. Mech.* 4, 575-579. 10.1242/dmm.007823 21810904 PMC3180220

[DMM052086C17] Bhatt, A. S., Jering, K. S., Vaduganathan, M., Claggett, B. L., Cunningham, J. W., Rosenthal, N., Signorovitch, J., Thune, J. J., Vardeny, O. and Solomon, S. D. (2021). Clinical outcomes in patients with heart failure hospitalized with COVID-19. *JACC Heart Fail.* 9, 65-73. 10.1016/j.jchf.2020.11.003 33384064 PMC7833294

[DMM052086C18] Bilinska, K., Von Bartheld, C. S. and Butowt, R. (2021). Expression of the ACE2 virus entry protein in the nervus terminalis reveals the potential for an alternative route to brain infection in COVID-19. *Front. Cell Neurosci.* 15, 674123. 10.3389/fncel.2021.674123 34290590 PMC8287262

[DMM052086C19] Blair, R. V., Vaccari, M., Doyle-Meyers, L. A., Roy, C. J., Russell-Lodrigue, K., Fahlberg, M., Monjure, C. J., Beddingfield, B., Plante, K. S., Plante, J. A. et al. (2021). Acute respiratory distress in aged, SARS-CoV-2-infected african green monkeys but not rhesus macaques. *Am. J. Pathol.* 191, 274-282. 10.1016/j.ajpath.2020.10.016 33171111 PMC7648506

[DMM052086C20] Bleau, C., Filliol, A., Samson, M. and Lamontagne, L. (2015). Brain invasion by mouse hepatitis virus depends on impairment of tight junctions and beta interferon production in brain microvascular endothelial cells. *J. Virol.* 89, 9896-9908. 10.1128/JVI.01501-15 26202229 PMC4577898

[DMM052086C21] Bosmuller, H., Matter, M., Fend, F. and Tzankov, A. (2021). The pulmonary pathology of COVID-19. *Virchows Arch.* 478, 137-150. 10.1007/s00428-021-03053-1 33604758 PMC7892326

[DMM052086C22] Boszormenyi, K. P., Stammes, M. A., Fagrouch, Z. C., Kiemenyi-Kayere, G., Niphuis, H., Mortier, D., Van Driel, N., Nieuwenhuis, I., Vervenne, R. A. W., Haaksma, T. et al. (2021). The post-acute phase of SARS-CoV-2 infection in two macaque species is associated with signs of ongoing virus replication and pathology in pulmonary and extrapulmonary tissues. *Viruses* 13, 1673. 10.3390/v13081673 34452537 PMC8402919

[DMM052086C23] Braniste, V., Al-Asmakh, M., Kowal, C., Anuar, F., Abbaspour, A., Tóth, M., Korecka, A., Bakocevic, N., Ng, L. G., Kundu, P. et al. (2014). The gut microbiota influences blood-brain barrier permeability in mice. *Sci. Transl. Med.* 6, 263ra158. 10.1126/scitranslmed.3009759 PMC4396848 25411471

[DMM052086C24] Bryche, B., St Albin, A., Murri, S., Lacôte, S., Pulido, C., Ar Gouilh, M., Lesellier, S., Servat, A., Wasniewski, M., Picard-Meyer, E. et al. (2020). Massive transient damage of the olfactory epithelium associated with infection of sustentacular cells by SARS-CoV-2 in golden Syrian hamsters. *Brain Behav. Immun.* 89, 579-586. 10.1016/j.bbi.2020.06.032 32629042 PMC7332942

[DMM052086C25] Bryda, E. C. (2013). The mighty mouse: the impact of rodents on advances in biomedical research. *Mo. Med.* 110, 207-211. 23829104 PMC3987984

[DMM052086C26] Cai, J., Lin, K., Zhang, H., Xue, Q., Zhu, K., Yuan, G., Sun, Y., Zhu, F., Ai, J. and Wang, S. et al. (2023). A one-year follow-up study of systematic impact of long COVID symptoms among patients post SARS-CoV-2 omicron variants infection in Shanghai, China. *Emerg. Microbes Infect.* 12, 2220578. 10.1080/22221751.2023.2220578 37272336 PMC10281439

[DMM052086C27] Calabrese, L. H., Winthrop, K., Strand, V., Yazdany, J. and Walter, J. E. (2021). Type I interferon, anti-interferon antibodies, and COVID-19. *Lancet Rheumatol.* 3, e246-e247. 10.1016/S2665-9913(21)00034-5 33655222 PMC7906736

[DMM052086C28] Carabotti, M., Scirocco, A., Maselli, M. A. and Severi, C. (2015). The gut-brain axis: interactions between enteric microbiota, central and enteric nervous systems. *Ann. Gastroenterol.* 28, 203-209. 25830558 PMC4367209

[DMM052086C29] Carnevale, S., Beretta, P. and Morbini, P. (2021). Direct endothelial damage and vasculitis due to SARS-CoV-2 in small bowel submucosa of COVID-19 patient with diarrhea. *J. Med. Virol.* 93, 61-63. 10.1002/jmv.26119 32492199 PMC7300801

[DMM052086C30] Carossino, M., Kenney, D., O'Connell, A. K., Montanaro, P., Tseng, A. E., Gertje, H. P., Grosz, K. A., Ericsson, M., Huber, B. R., Kurnick, S. A. et al. (2022). Fatal neurodissemination and SARS-CoV-2 tropism in K18-hACE2 mice is only partially dependent on hACE2 expression. *Viruses* 14, 535. 10.3390/v14030535 35336942 PMC8955233

[DMM052086C31] Carpenter, K. C., Yang, J. and Xu, J. J. (2023). Animal models for the study of neurologic manifestations of COVID-19. *Comp. Med.* 73, 91-103. 10.30802/AALAS-CM-22-000073 36744556 PMC9948905

[DMM052086C32] Cascella, M., Rajnik, M., Aleem, A., Dulebohn, S. C. and Di Napoli, R. (2023). *Features, Evaluation, and Treatment of Coronavirus (COVID-19)*. Treasure Island, FL: StatPearls. 32150360

[DMM052086C33] Chen, Y., Chen, L., Deng, Q., Zhang, G., Wu, K., Ni, L., Yang, Y., Liu, B., Wang, W., Wei, C. et al. (2020). The presence of SARS-CoV-2 RNA in the feces of COVID-19 patients. *J. Med. Virol.* 92, 833-840. 10.1002/jmv.25825 32243607

[DMM052086C219] Chen, Q., Huang, X.-Y., Liu, Y., Sun, M.-X., Ji, B., Zhou, C., Chi, H., Zhang, R.-R., Luo, D., Tian, Y. et al. (2022). Comparative characterization of SARS-CoV-2 variants of concern and mouse-adapted strains in mice. *J. Med. Virol.* 94, 3223-3232. 10.1002/jmv.27735 35322439 PMC9088695

[DMM052086C34] Choudhary, S., Kanevsky, I., Yildiz, S., Sellers, R. S., Swanson, K. A., Franks, T., Rathnasinghe, R., Munoz-Moreno, R., Jangra, S., Gonzalez, O. et al. (2022). Modeling SARS-CoV-2: comparative pathology in rhesus macaque and golden syrian hamster models. *Toxicol. Pathol.* 50, 280-293. 10.1177/01926233211072767 35128980 PMC8819578

[DMM052086C35] Ciurkiewicz, M., Armando, F., Schreiner, T., De Buhr, N., Pilchová, V., Krupp-Buzimikic, V., Gabriel, G., Von Köckritz-Blickwede, M., Baumgärtner, W., Schulz, C. et al. (2022). Ferrets are valuable models for SARS-CoV-2 research. *Vet. Pathol.* 59, 661-672. 10.1177/03009858211071012 35001763 PMC9207987

[DMM052086C36] Clerbaux, L. A., Fillipovska, J., Muñoz, A., Petrillo, M., Coecke, S., Amorim, M. J. and Grenga, L. (2022). Mechanisms leading to gut Dysbiosis in COVID-19: current evidence and uncertainties based on adverse outcome pathways. *J Clin. Med.* 11, 5400. 10.3390/jcm11185400 36143044 PMC9505288

[DMM052086C37] Cojocaru, E., Cojocaru, C., Vlad, C. E. and Eva, L. (2023). Role of the renin-angiotensin system in long COVID's cardiovascular injuries. *Biomedicines* 11, 2004. 10.3390/biomedicines11072004 37509643 PMC10377338

[DMM052086C38] Connors, J. M. and Levy, J. H. (2020). COVID-19 and its implications for thrombosis and anticoagulation. *Blood* 135, 2033-2040. 10.1182/blood.2020006000 32339221 PMC7273827

[DMM052086C39] Coto, E., Avanzas, P. and Gómez, J. (2021). The renin-angiotensin-aldosterone system and coronavirus disease 2019. *Eur. Cardiol.* 16, e07. 10.15420/ecr.2020.30 33737961 PMC7967817

[DMM052086C40] Craddock, V., Mahajan, A., Spikes, L., Krishnamachary, B., Ram, A. K., Kumar, A., Chen, L., Chalise, P. and Dhillon, N. K. (2023). Persistent circulation of soluble and extracellular vesicle-linked Spike protein in individuals with postacute sequelae of COVID-19. *J. Med. Virol.* 95, e28568. 10.1002/jmv.28568 36756925 PMC10048846

[DMM052086C41] Cross, R. W., Agans, K. N., Prasad, A. N., Borisevich, V., Woolsey, C., Deer, D. J., Dobias, N. S., Geisbert, J. B., Fenton, K. A. and Geisbert, T. W. (2020). Intranasal exposure of African green monkeys to SARS-CoV-2 results in acute phase pneumonia with shedding and lung injury still present in the early convalescence phase. *Virol. J.* 17, 125. 10.1186/s12985-020-01396-w 32811514 PMC7431901

[DMM052086C42] Dan, S., Pant, M. and Upadhyay, S. K. (2020). The case fatality rate in COVID-19 patients with cardiovascular disease: global health challenge and paradigm in the current pandemic. *Curr. Pharmacol. Rep.* 6, 315-324. 10.1007/s40495-020-00239-0 32953401 PMC7490208

[DMM052086C43] Daugherty, S. E., Guo, Y., Heath, K., Dasmarinas, M. C., Jubilo, K. G., Samranvedhya, J., Lipsitch, M. and Cohen, K. (2021). Risk of clinical sequelae after the acute phase of SARS-CoV-2 infection: retrospective cohort study. *BMJ* 373, n1098. 10.1136/bmj.n1098 34011492 PMC8132065

[DMM052086C44] De Melo, G. D., Lazarini, F., Levallois, S., Hautefort, C., Michel, V., Larrous, F., Verillaud, B., Aparicio, C., Wagner, S., Gheusi, G. et al. (2021). COVID-19-related anosmia is associated with viral persistence and inflammation in human olfactory epithelium and brain infection in hamsters. *Sci. Transl. Med.* 13, eabf8396. 10.1126/scitranslmed.abf8396 33941622 PMC8158965

[DMM052086C45] De Oliveira, A. P., Lopes, A. L. F., Pacheco, G., De Sá Guimarães Nolêto, I. R., Nicolau, L. A. D. and Medeiros, J. V. R. (2020). Premises among SARS-CoV-2, dysbiosis and diarrhea: walking through the ACE2/mTOR/autophagy route. *Med. Hypotheses* 144, 110243. 10.1016/j.mehy.2020.110243 33254549 PMC7467124

[DMM052086C46] De Wit, E., Rasmussen, A. L., Falzarano, D., Bushmaker, T., Feldmann, F., Brining, D. L., Fischer, E. R., Martellaro, C., Okumura, A., Chang, J. et al. (2013). Middle East respiratory syndrome coronavirus (MERS-CoV) causes transient lower respiratory tract infection in rhesus macaques. *Proc. Natl. Acad. Sci. USA* 110, 16598-16603. 10.1073/pnas.1310744110 24062443 PMC3799368

[DMM052086C47] Din, A. U., Mazhar, M., Waseem, M., Ahmad, W., Bibi, A., Hassan, A., Ali, N., Gang, W., Qian, G., Ullah, R. et al. (2021). SARS-CoV-2 microbiome dysbiosis linked disorders and possible probiotics role. *Biomed. Pharmacother.* 133, 110947. 10.1016/j.biopha.2020.110947 33197765 PMC7657099

[DMM052086C48] Dinnon, K. H., 3rd, Leist, S. R., Schafer, A., Edwards, C. E., Martinez, D. R., Montgomery, S. A., West, A., Yount, B. L., Jr, Hou, Y. J., Adams, L. E. et al. (2020). A mouse-adapted model of SARS-CoV-2 to test COVID-19 countermeasures. *Nature* 586, 560-566. 10.1038/s41586-020-2708-8 32854108 PMC8034761

[DMM052086C49] Dinnon, K. H., 3rd, Leist, S. R., Okuda, K., Dang, H., Fritch, E. J., Gully, K. L., De La Cruz, G., Evangelista, M. D., Asakura, T., Gilmore, R. C. et al. (2022). SARS-CoV-2 infection produces chronic pulmonary epithelial and immune cell dysfunction with fibrosis in mice. *Sci. Transl. Med.* 14, eabo5070. 10.1126/scitranslmed.abo5070 35857635 PMC9273046

[DMM052086C50] Dong, W., Mead, H., Tian, L., Park, J. G., Garcia, J. I., Jaramillo, S., Barr, T., Kollath, D. S., Coyne, V. K., Stone, N. E. et al. (2022). The K18-human ACE2 transgenic mouse model recapitulates non-severe and severe COVID-19 in response to an infectious dose of the SARS-CoV-2 virus. *J. Virol.* 96, e0096421. 34668775 10.1128/JVI.00964-21 PMC8754221

[DMM052086C51] Ellul, M. A., Benjamin, L., Singh, B., Lant, S., Michael, B. D., Easton, A., Kneen, R., Defres, S., Sejvar, J. and Solomon, T. (2020). Neurological associations of COVID-19. *Lancet Neurol.* 19, 767-783. 10.1016/S1474-4422(20)30221-0 32622375 PMC7332267

[DMM052086C52] Elmakaty, I., Ferih, K., Karen, O., Ouda, A., Elsabagh, A., Amarah, A. and Malki, M. I. (2022). Clinical implications of COVID-19 presence in CSF: systematic review of case reports. *Cells* 11, 3212. 10.3390/cells11203212 36291083 PMC9600635

[DMM052086C53] Erny, D., Hrabě De Angelis, A. L., Jaitin, D., Wieghofer, P., Staszewski, O., David, E., Keren-Shaul, H., Mahlakoiv, T., Jakobshagen, K., Buch, T. et al. (2015). Host microbiota constantly control maturation and function of microglia in the CNS. *Nat. Neurosci.* 18, 965-977. 10.1038/nn.4030 26030851 PMC5528863

[DMM052086C54] Everett, H. E., Lean, F. Z. X., Byrne, A. M. P., Van Diemen, P. M., Rhodes, S., James, J., Mollett, B., Coward, V. J., Skinner, P., Warren, C. J. et al. (2021). Intranasal infection of ferrets with SARS-CoV-2 as a model for asymptomatic human infection. *Viruses* 13, 113. 10.3390/v13010113 33467732 PMC7830262

[DMM052086C55] Feng, Y., Song, X., Huang, Y., Deng, W., Li, M., Guo, X., Qin, C., Tong, W. M., Liu, J. and Wang, J. (2021). SARS-CoV-2 leads to myocardial injury in rhesus macaque. *Signal Transduct. Target. Ther.* 6, 338. 10.1038/s41392-021-00747-5 34489396 PMC8419658

[DMM052086C56] Fontes-Dantas, F. L., Fernandes, G. G., Gutman, E. G., De Lima, E. V., Antonio, L. S., Hammerle, M. B., Mota-Araujo, H. P., Colodeti, L. C., Araújo, S. M. B., Froz, G. M. et al. (2023). SARS-CoV-2 Spike protein induces TLR4-mediated long-term cognitive dysfunction recapitulating post-COVID-19 syndrome in mice. *Cell Rep.* 42, 112189. 10.1016/j.celrep.2023.112189 36857178 PMC9935273

[DMM052086C57] Francis, M. E., Goncin, U., Kroeker, A., Swan, C., Ralph, R., Lu, Y., Etzioni, A. L., Falzarano, D., Gerdts, V., Machtaler, S. et al. (2021). SARS-CoV-2 infection in the Syrian hamster model causes inflammation as well as type I interferon dysregulation in both respiratory and non-respiratory tissues including the heart and kidney. *PLoS Pathog.* 17, e1009705. 10.1371/journal.ppat.1009705 34265022 PMC8282065

[DMM052086C58] Frere, J. J., Serafini, R. A., Pryce, K. D., Zazhytska, M., Oishi, K., Golynker, I., Panis, M., Zimering, J., Horiuchi, S., Hoagland, D. A. et al. (2022). SARS-CoV-2 infection in hamsters and humans results in lasting and unique systemic perturbations after recovery. *Sci. Transl. Med.* 14, eabq3059. 10.1126/scitranslmed.abq3059 35857629 PMC9210449

[DMM052086C59] Fumagalli, V., Ravà, M., Marotta, D., Di Lucia, P., Laura, C., Sala, E., Grillo, M., Bono, E., Giustini, L., Perucchini, C. et al. (2022). Administration of aerosolized SARS-CoV-2 to K18-hACE2 mice uncouples respiratory infection from fatal neuroinvasion. *Sci. Immunol.* 7, eabl9929. 10.1126/sciimmunol.abl9929 34812647 PMC9835999

[DMM052086C60] García-Azorín, D., Martínez-Pías, E., Trigo, J., Hernández-Pérez, I., Valle-Peñacoba, G., Talavera, B., Simón-Campo, P., De Lera, M., Chavarría-Miranda, A., López-Sanz, C. et al. (2020). Neurological comorbidity is a predictor of death in covid-19 disease: a cohort study on 576 patients. *Front. Neurol.* 11, 781. 10.3389/fneur.2020.00781 32733373 PMC7358573

[DMM052086C61] Geca, T., Wojtowicz, K., Guzik, P. and Gora, T. (2022). Increased risk of COVID-19 in patients with diabetes mellitus-current challenges in pathophysiology, treatment and prevention. *Int. J. Environ. Res. Public Health* 19, 6555. 10.3390/ijerph19116555 35682137 PMC9180541

[DMM052086C62] Giamarellos-Bourboulis, E. J., Netea, M. G., Rovina, N., Akinosoglou, K., Antoniadou, A., Antonakos, N., Damoraki, G., Gkavogianni, T., Adami, M. E., Katsaounou, P. et al. (2020). Complex immune dysregulation in COVID-19 patients with severe respiratory failure. *Cell Host Microbe* 27, 992-1000.e3. 10.1016/j.chom.2020.04.009 32320677 PMC7172841

[DMM052086C63] Golden, J. W., Cline, C. R., Zeng, X., Garrison, A. R., Carey, B. D., Mucker, E. M., White, L. E., Shamblin, J. D., Brocato, R. L., Liu, J. et al. (2020). Human angiotensin-converting enzyme 2 transgenic mice infected with SARS-CoV-2 develop severe and fatal respiratory disease. *JCI Insight* 5, e142032. 10.1172/jci.insight.142032 32841215 PMC7566707

[DMM052086C64] Golden, J. W., Li, R., Cline, C. R., Zeng, X., Mucker, E. M., Fuentes-Lao, A. J., Spik, K. W., Williams, J. A., Twenhafel, N., Davis, N. et al. (2022). Hamsters expressing human angiotensin-converting enzyme 2 develop severe disease following exposure to SARS-CoV-2. *mBio* 13, e0290621. 10.1128/mbio.02906-21 35073750 PMC8787465

[DMM052086C65] Goncalves, A., Maisonnasse, P., Donati, F., Albert, M., Behillil, S., Contreras, V., Naninck, T., Marlin, R., Solas, C., Pizzorno, A. et al. (2021). SARS-CoV-2 viral dynamics in non-human primates. *PLoS Comput. Biol.* 17, e1008785. 10.1371/journal.pcbi.1008785 33730053 PMC8007039

[DMM052086C66] Gressett, T. E., Leist, S. R., Ismael, S., Talkington, G., Dinnon, K. H., Baric, R. S. and Bix, G. (2023). Mouse adapted SARS-CoV-2 model induces “long-COVID” neuropathology in BALB/c mice. *bioRxiv*.

[DMM052086C67] Gu, S., Chen, Y., Wu, Z., Chen, Y., Gao, H., Lv, L., Guo, F., Zhang, X., Luo, R., Huang, C. et al. (2020). Alterations of the gut microbiota in patients with coronavirus disease 2019 or H1N1 influenza. *Clin. Infect. Dis.* 71, 2669-2678. 10.1093/cid/ciaa709 32497191 PMC7314193

[DMM052086C68] Hart, M. L., Ericsson, A. C., Lloyd, K. C. K., Grimsrud, K. N., Rogala, A. R., Godfrey, V. L., Nielsen, J. N. and Franklin, C. L. (2018). Development of outbred CD1 mouse colonies with distinct standardized gut microbiota profiles for use in complex microbiota targeted studies. *Sci. Rep.* 8, 10107. 10.1038/s41598-018-28448-0 29973630 PMC6031694

[DMM052086C69] Hartman, A. L., Nambulli, S., McMillen, C. M., White, A. G., Tilston-Lunel, N. L., Albe, J. R., Cottle, E., Dunn, M. D., Frye, L. J., Gilliland, T. H. et al. (2020). SARS-CoV-2 infection of African green monkeys results in mild respiratory disease discernible by PET/CT imaging and shedding of infectious virus from both respiratory and gastrointestinal tracts. *PLoS Pathog.* 16, e1008903. 10.1371/journal.ppat.1008903 32946524 PMC7535860

[DMM052086C70] Hernandez-Romieu, A. C., Carton, T. W., Saydah, S., Azziz-Baumgartner, E., Boehmer, T. K., Garret, N. Y., Bailey, L. C., Cowell, L. G., Draper, C., Mayer, K. H. et al. (2022). Prevalence of select new symptoms and conditions among persons aged younger than 20 years and 20 years or older at 31 to 150 days after testing positive or negative for SARS-CoV-2. *JAMA Netw. Open* 5, e2147053. 10.1001/jamanetworkopen.2021.47053 35119459 PMC8817203

[DMM052086C71] Hoffmann, M., Kleine-Weber, H., Schroeder, S., Kruger, N., Herrler, T., Erichsen, S., Schiergens, T. S., Herrler, G., Wu, N. H., Nitsche, A. et al. (2020). SARS-CoV-2 cell entry depends on ACE2 and TMPRSS2 and is blocked by a clinically proven protease inhibitor. *Cell* 181, 271-280.e8. 10.1016/j.cell.2020.02.052 32142651 PMC7102627

[DMM052086C72] Huang, S., Wang, J., Liu, F., Liu, J., Cao, G., Yang, C., Liu, W., Tu, C., Zhu, M. and Xiong, B. (2020). COVID-19 patients with hypertension have more severe disease: a multicenter retrospective observational study. *Hypertens. Res.* 43, 824-831. 10.1038/s41440-020-0485-2 32483311 PMC7261650

[DMM052086C73] Iba, T., Connors, J. M. and Levy, J. H. (2020). The coagulopathy, endotheliopathy, and vasculitis of COVID-19. *Inflamm. Res.* 69, 1181-1189. 10.1007/s00011-020-01401-6 32918567 PMC7486586

[DMM052086C74] Imai, M., Iwatsuki-Horimoto, K., Hatta, M., Loeber, S., Halfmann, P. J., Nakajima, N., Watanabe, T., Ujie, M., Takahashi, K., Ito, M. et al. (2020). Syrian hamsters as a small animal model for SARS-CoV-2 infection and countermeasure development. *Proc. Natl. Acad. Sci. USA* 117, 16587-16595. 10.1073/pnas.2009799117 32571934 PMC7368255

[DMM052086C75] Iwata-Yoshikawa, N., Kakizaki, M., Shiwa-Sudo, N., Okura, T., Tahara, M., Fukushi, S., Maeda, K., Kawase, M., Asanuma, H., Tomita, Y. et al. (2022). Essential role of TMPRSS2 in SARS-CoV-2 infection in murine airways. *Nat. Commun.* 13, 6100. 10.1038/s41467-022-33911-8 36243815 PMC9568946

[DMM052086C76] Jacobs, J. L., Bain, W., Naqvi, A., Staines, B., Castanha, P. M. S., Yang, H., Boltz, V. F., Barratt-Boyes, S., Marques, E. T. A., Mitchell, S. L. et al. (2022). Severe acute respiratory syndrome coronavirus 2 viremia is associated with coronavirus disease 2019 severity and predicts clinical outcomes. *Clin. Infect. Dis.* 74, 1525-1533. 10.1093/cid/ciab686 34374761 PMC9070832

[DMM052086C77] Jansen, E. B., Orvold, S. N., Swan, C. L., Yourkowski, A., Thivierge, B. M., Francis, M. E., Ge, A., Rioux, M., Darbellay, J., Howland, J. G. et al. (2022). After the virus has cleared-Can preclinical models be employed for Long COVID research? *PLoS Pathog.* 18, e1010741. 10.1371/journal.ppat.1010741 36070309 PMC9451097

[DMM052086C78] Jiang, R. D., Liu, M. Q., Chen, Y., Shan, C., Zhou, Y. W., Shen, X. R., Li, Q., Zhang, L., Zhu, Y., Si, H. R. et al. (2020). Pathogenesis of SARS-CoV-2 in transgenic mice expressing human angiotensin-converting enzyme 2. *Cell* 182, 50-58.e8. 10.1016/j.cell.2020.05.027 32516571 PMC7241398

[DMM052086C79] Jiao, L., Yang, Y., Yu, W., Zhao, Y., Long, H., Gao, J., Ding, K., Ma, C., Li, J., Zhao, S. et al. (2021). The olfactory route is a potential way for SARS-CoV-2 to invade the central nervous system of rhesus monkeys. *Signal Transduct. Target. Ther.* 6, 169. 10.1038/s41392-021-00591-7 33895780 PMC8065334

[DMM052086C80] Jin, X., Lian, J. S., Hu, J. H., Gao, J., Zheng, L., Zhang, Y. M., Hao, S. R., Jia, H. Y., Cai, H., Zhang, X. L. et al. (2020). Epidemiological, clinical and virological characteristics of 74 cases of coronavirus-infected disease 2019 (COVID-19) with gastrointestinal symptoms. *Gut* 69, 1002-1009. 10.1136/gutjnl-2020-320926 32213556 PMC7133387

[DMM052086C81] Joffre, J., Rodriguez, L., Matthay, Z. A., Lloyd, E., Fields, A. T., Bainton, R. J., Kurien, P., Sil, A., Calfee, C. S., Woodruff, P. G. et al. (2022). COVID-19-associated lung microvascular endotheliopathy: a “from the bench” perspective. *Am. J. Respir. Crit. Care. Med.* 206, 961-972. 10.1164/rccm.202107-1774OC 35649173 PMC9801996

[DMM052086C82] Käufer, C., Schreiber, C. S., Hartke, A. S., Denden, I., Stanelle-Bertram, S., Beck, S., Kouassi, N. M., Beythien, G., Becker, K., Schreiner, T. et al. (2022). Microgliosis and neuronal proteinopathy in brain persist beyond viral clearance in SARS-CoV-2 hamster model. *EBioMedicine* 79, 103999. 10.1016/j.ebiom.2022.103999 35439679 PMC9013202

[DMM052086C83] Ketcham, S. W., Bolig, T. C., Molling, D. J., Sjoding, M. W., Flanders, S. A. and Prescott, H. C. (2021). Causes and circumstances of death among patients hospitalized with COVID-19: a retrospective cohort study. *Ann. Am. Thorac. Soc.* 18, 1076-1079. 10.1513/AnnalsATS.202011-1381RL 33315531 PMC8456727

[DMM052086C84] Khan, I., Bai, Y., Zha, L., Ullah, N., Ullah, H., Shah, S. R. H., Sun, H. and Zhang, C. (2021). Mechanism of the gut microbiota colonization resistance and enteric pathogen infection. *Front. Cell Infect. Microbiol.* 11, 716299. 10.3389/fcimb.2021.716299 35004340 PMC8733563

[DMM052086C85] Kim, Y. I., Kim, S. G., Kim, S. M., Kim, E. H., Park, S. J., Yu, K. M., Chang, J. H., Kim, E. J., Lee, S., Casel, M. A. B. et al. (2020). Infection and rapid transmission of SARS-CoV-2 in ferrets. *Cell Host Microbe* 27, 704-709.e2. 10.1016/j.chom.2020.03.023 32259477 PMC7144857

[DMM052086C86] Kinnebrew, M. A., Ubeda, C., Zenewicz, L. A., Smith, N., Flavell, R. A. and Pamer, E. G. (2010). Bacterial flagellin stimulates Toll-like receptor 5-dependent defense against vancomycin-resistant Enterococcus infection. *J. Infect. Dis.* 201, 534-543. 10.1086/650203 20064069 PMC2811237

[DMM052086C87] Kuiken, T., Fouchier, R. A., Schutten, M., Rimmelzwaan, G. F., Van Amerongen, G., Van Riel, D., Laman, J. D., De Jong, T., Van Doornum, G., Lim, W. et al. (2003). Newly discovered coronavirus as the primary cause of severe acute respiratory syndrome. *Lancet* 362, 263-270. 10.1016/S0140-6736(03)13967-0 12892955 PMC7112434

[DMM052086C88] Kumari, P., Rothan, H. A., Natekar, J. P., Stone, S., Pathak, H., Strate, P. G., Arora, K., Brinton, M. A. and Kumar, M. (2021). Neuroinvasion and encephalitis following intranasal inoculation of SARS-CoV-2 in K18-hACE2 mice. *Viruses* 13, 132. 10.3390/v13010132 33477869 PMC7832889

[DMM052086C89] Kutter, J. S., De Meulder, D., Bestebroer, T. M., Lexmond, P., Mulders, A., Richard, M., Fouchier, R. A. M. and Herfst, S. (2021). SARS-CoV and SARS-CoV-2 are transmitted through the air between ferrets over more than one meter distance. *Nat. Commun.* 12, 1653. 10.1038/s41467-021-21918-6 33712573 PMC7955093

[DMM052086C90] Laboratory, J. (2023). *K18-hACE2 Strain Details [Online]*. Jax Laboratory. Available: https://www.jax.org/strain/034860 [Accessed 2023].

[DMM052086C91] Lamers, M. M., Beumer, J., Van Der Vaart, J., Knoops, K., Puschhof, J., Breugem, T. I., Ravelli, R. B. G., Paul Van Schayck, J., Mykytyn, A. Z., Duimel, H. Q. et al. (2020). SARS-CoV-2 productively infects human gut enterocytes. *Science* 369, 50-54. 10.1126/science.abc1669 32358202 PMC7199907

[DMM052086C92] Lamoureux, L., Sajesh, B., Slota, J. A., Medina, S. J., Mayor, M., Frost, K. L., Warner, B., Manguiat, K., Wood, H., Kobasa, D. et al. (2022). Non-productive infection of glial cells with SARS-CoV-2 in hamster organotypic cerebellar slice cultures. *Viruses* 14, 1218. 10.3390/v14061218 35746689 PMC9227386

[DMM052086C93] Lawler, J. V., Endy, T. P., Hensley, L. E., Garrison, A., Fritz, E. A., Lesar, M., Baric, R. S., Kulesh, D. A., Norwood, D. A., Wasieloski, L. P. et al. (2006). Cynomolgus macaque as an animal model for severe acute respiratory syndrome. *PLoS Med.* 3, e149. 10.1371/journal.pmed.0030149 16605302 PMC1435788

[DMM052086C94] Lehmann, M., Allers, K., Heldt, C., Meinhardt, J., Schmidt, F., Rodriguez-Sillke, Y., Kunkel, D., Schumann, M., Böttcher, C., Stahl-Hennig, C. et al. (2021). Human small intestinal infection by SARS-CoV-2 is characterized by a mucosal infiltration with activated CD8(+) T cells. *Mucosal. Immunol.* 14, 1381-1392. 10.1038/s41385-021-00437-z 34420043 PMC8379580

[DMM052086C95] Leist, S. R., Dinnon, K. H., III, Schafer, A., Tse, L. V., Okuda, K., Hou, Y. J., West, A., Edwards, C. E., Sanders, W., Fritch, E. J. et al. (2020). A mouse-adapted SARS-CoV-2 induces acute lung injury and mortality in standard laboratory mice. *Cell* 183, 1070-1085.e12. 10.1016/j.cell.2020.09.050 33031744 PMC7510428

[DMM052086C96] Letsinger, A. C., Ward, J. M., Fannin, R. D., Mahapatra, D., Bridge, M. F., Sills, R. C., Gerrish, K. E. and Yakel, J. L. (2023). Nicotine exposure decreases likelihood of SARS-CoV-2 RNA expression and neuropathology in the hACE2 mouse brain but not moribundity. *Sci. Rep.* 13, 2042. 10.1038/s41598-023-29118-6 36739463 PMC9898857

[DMM052086C97] Levi, M., Thachil, J., Iba, T. and Levy, J. H. (2020). Coagulation abnormalities and thrombosis in patients with COVID-19. *Lancet Haematol.* 7, e438-e440. 10.1016/S2352-3026(20)30145-9 32407672 PMC7213964

[DMM052086C98] Li, N., Ma, W. T., Pang, M., Fan, Q. L. and Hua, J. L. (2019). The commensal microbiota and viral infection: a comprehensive review. *Front. Immunol.* 10, 1551. 10.3389/fimmu.2019.01551 31333675 PMC6620863

[DMM052086C99] Li, Z., Liu, T., Yang, N., Han, D., Mi, X., Li, Y., Liu, K., Vuylsteke, A., Xiang, H. and Guo, X. (2020). Neurological manifestations of patients with COVID-19: potential routes of SARS-CoV-2 neuroinvasion from the periphery to the brain. *Front. Med.* 14, 533-541. 10.1007/s11684-020-0786-5 32367431 PMC7197033

[DMM052086C100] Liotta, E. M., Batra, A., Clark, J. R., Shlobin, N. A., Hoffman, S. C., Orban, Z. S. and Koralnik, I. J. (2020). Frequent neurologic manifestations and encephalopathy-associated morbidity in Covid-19 patients. *Ann. Clin. Transl. Neurol.* 7, 2221-2230. 10.1002/acn3.51210 33016619 PMC7664279

[DMM052086C101] Littlefield, K. M., Watson, R. O., Schneider, J. M., Neff, C. P., Yamada, E., Zhang, M., Campbell, T. B., Falta, M. T., Jolley, S. E., Fontenot, A. P. et al. (2022). SARS-CoV-2-specific T cells associate with inflammation and reduced lung function in pulmonary post-acute sequalae of SARS-CoV-2. *PLoS Pathog.* 18, e1010359. 10.1371/journal.ppat.1010359 35617421 PMC9176759

[DMM052086C102] Liu, F., Liu, F. and Wang, L. (2021). COVID-19 and cardiovascular diseases. *J. Mol. Cell Biol.* 13, 161-167. 10.1093/jmcb/mjaa064 33226078 PMC7717280

[DMM052086C103] Liu, J., Lu, F., Chen, Y., Plow, E. and Qin, J. (2022a). Integrin mediates cell entry of the SARS-CoV-2 virus independent of cellular receptor ACE2. *J. Biol. Chem.* 298, 101710. 10.1016/j.jbc.2022.101710 35150743 PMC8828381

[DMM052086C104] Liu, Q., Mak, J. W. Y., Su, Q., Yeoh, Y. K., Lui, G. C., Ng, S. S. S., Zhang, F., Li, A. Y. L., Lu, W., Hui, D. S. et al. (2022b). Gut microbiota dynamics in a prospective cohort of patients with post-acute COVID-19 syndrome. *Gut* 71, 544-552. 10.1136/gutjnl-2021-325989 35082169

[DMM052086C105] Lou, J. J., Movassaghi, M., Gordy, D., Olson, M. G., Zhang, T., Khurana, M. S., Chen, Z., Perez-Rosendahl, M., Thammachantha, S., Singer, E. J. et al. (2021). Neuropathology of COVID-19 (neuro-COVID): clinicopathological update. *Free. Neuropathol.* 2, 2. 10.17879/freeneuropathology-2021-2993 33554218 PMC7861505

[DMM052086C106] Luan, J., Lu, Y., Jin, X. and Zhang, L. (2020). Spike protein recognition of mammalian ACE2 predicts the host range and an optimized ACE2 for SARS-CoV-2 infection. *Biochem. Biophys. Res. Commun.* 526, 165-169. 10.1016/j.bbrc.2020.03.047 32201080 PMC7102515

[DMM052086C107] Manca, C., Boubertakh, B., Leblanc, N., Deschênes, T., Lacroix, S., Martin, C., Houde, A., Veilleux, A., Flamand, N., Muccioli, G. G. et al. (2020). Germ-free mice exhibit profound gut microbiota-dependent alterations of intestinal endocannabinoidome signaling. *J. Lipid Res.* 61, 70-85. 10.1194/jlr.RA119000424 31690638 PMC6939599

[DMM052086C108] Mantovani, S., Oliviero, B., Varchetta, S., Renieri, A. and Mondelli, M. U. (2023). TLRs: innate immune sentries against SARS-CoV-2 infection. *Int. J. Mol. Sci.* 24, 8065. 10.3390/ijms24098065 37175768 PMC10178469

[DMM052086C109] Mao, L., Jin, H., Wang, M., Hu, Y., Chen, S., He, Q., Chang, J., Hong, C., Zhou, Y., Wang, D. et al. (2020a). Neurologic manifestations of hospitalized patients with coronavirus disease 2019 in Wuhan, China. *JAMA Neurol.* 77, 683-690. 10.1001/jamaneurol.2020.1127 32275288 PMC7149362

[DMM052086C110] Mao, R., Qiu, Y., He, J. S., Tan, J. Y., Li, X. H., Liang, J., Shen, J., Zhu, L. R., Chen, Y., Iacucci, M. et al. (2020b). Manifestations and prognosis of gastrointestinal and liver involvement in patients with COVID-19: a systematic review and meta-analysis. *Lancet Gastroenterol. Hepatol.* 5, 667-678. 10.1016/S2468-1253(20)30126-6 32405603 PMC7217643

[DMM052086C111] Martinez-Salazar, B., Holwerda, M., Studle, C., Piragyte, I., Mercader, N., Engelhardt, B., Rieben, R. and Doring, Y. (2022). COVID-19 and the vasculature: current aspects and long-term consequences. *Front. Cell Dev. Biol.* 10, 824851. 10.3389/fcell.2022.824851 35242762 PMC8887620

[DMM052086C112] Mazmanian, S. K., Liu, C. H., Tzianabos, A. O. and Kasper, D. L. (2005). An immunomodulatory molecule of symbiotic bacteria directs maturation of the host immune system. *Cell* 122, 107-118. 10.1016/j.cell.2005.05.007 16009137

[DMM052086C113] McAuliffe, J., Vogel, L., Roberts, A., Fahle, G., Fischer, S., Shieh, W. J., Butler, E., Zaki, S., St Claire, M., Murphy, B. et al. (2004). Replication of SARS coronavirus administered into the respiratory tract of African Green, rhesus and cynomolgus monkeys. *Virology* 330, 8-15. 10.1016/j.virol.2004.09.030 15527829 PMC7111808

[DMM052086C114] McCray, P. B., Jr, Pewe, L., Wohlford-Lenane, C., Hickey, M., Manzel, L., Shi, L., Netland, J., Jia, H. P., Halabi, C., Sigmund, C. D. et al. (2007). Lethal infection of K18-hACE2 mice infected with severe acute respiratory syndrome coronavirus. *J. Virol.* 81, 813-821. 10.1128/JVI.02012-06 17079315 PMC1797474

[DMM052086C115] McDonald, D., Ackermann, G., Khailova, L., Baird, C., Heyland, D., Kozar, R., Lemieux, M., Derenski, K., King, J., Vis-Kampen, C. et al. (2016). Extreme dysbiosis of the microbiome in critical illness. *mSphere* 1, e00199-16. 10.1128/mSphere.00199-16 27602409 PMC5007431

[DMM052086C116] Meinhardt, J., Radke, J., Dittmayer, C., Franz, J., Thomas, C., Mothes, R., Laue, M., Schneider, J., Brünink, S., Greuel, S. et al. (2021). Olfactory transmucosal SARS-CoV-2 invasion as a port of central nervous system entry in individuals with COVID-19. *Nat. Neurosci.* 24, 168-175. 10.1038/s41593-020-00758-5 33257876

[DMM052086C117] Menachery, V. D., Yount, B. L., Jr., Sims, A. C., Debbink, K., Agnihothram, S. S., Gralinski, L. E., Graham, R. L., Scobey, T., Plante, J. A., Royal, S. R. et al. (2016). SARS-like WIV1-CoV poised for human emergence. *Proc. Natl. Acad. Sci. USA* 113, 3048-3053. 10.1073/pnas.1517719113 26976607 PMC4801244

[DMM052086C118] Merkler, A. E., Parikh, N. S., Mir, S., Gupta, A., Kamel, H., Lin, E., Lantos, J., Schenck, E. J., Goyal, P., Bruce, S. S. et al. (2020). Risk of ischemic stroke in patients with coronavirus disease 2019 (COVID-19) vs patients with influenza. *JAMA Neurol.* 77, 1-7. 10.1001/jamaneurol.2020.2730 32614385 PMC7333175

[DMM052086C119] Miao, J., Chard, L. S., Wang, Z. and Wang, Y. (2019). Syrian hamster as an animal model for the study on infectious diseases. *Front. Immunol.* 10, 2329. 10.3389/fimmu.2019.02329 31632404 PMC6781508

[DMM052086C120] Mohandas, S., Jain, R., Yadav, P. D., Shete-Aich, A., Sarkale, P., Kadam, M., Kumar, A., Deshpande, G., Baradkar, S., Patil, S. et al. (2020). Evaluation of the susceptibility of mice & hamsters to SARS-CoV-2 infection. *Indian J. Med. Res.* 151, 479-482. 10.4103/ijmr.IJMR_2235_20 32611917 PMC7530454

[DMM052086C121] Monchatre-Leroy, E., Lesellier, S., Wasniewski, M., Picard-Meyer, E., Richomme, C., Boué, F., Lacôte, S., Murri, S., Pulido, C., Vulin, J. et al. (2021). Hamster and ferret experimental infection with intranasal low dose of a single strain of SARS-CoV-2. *J. Gen. Virol.* 102, 001567. 10.1099/jgv.0.001567 33612147 PMC8515860

[DMM052086C122] Mouse Genome Sequencing Consortium, E. A. (2002). Initial sequencing and comparative analysis of the mouse genome. *Nature* 420, 520-562. 10.1038/nature01262 12466850

[DMM052086C123] Natarajan, A., Zlitni, S., Brooks, E. F., Vance, S. E., Dahlen, A., Hedlin, H., Park, R. M., Han, A., Schmidtke, D. T., Verma, R. et al. (2022). Gastrointestinal symptoms and fecal shedding of SARS-CoV-2 RNA suggest prolonged gastrointestinal infection. *Med.* 3, 371-387.e9. 10.1016/j.medj.2022.04.001 35434682 PMC9005383

[DMM052086C124] Ng, J. H., Sun, A., Je, H. S. and Tan, E. K. (2023). Unravelling Pathophysiology of Neurological and Psychiatric Complications of COVID-19 Using Brain Organoids. *Neuroscientist* 29, 30-40. 10.1177/10738584211015136 34036855 PMC9902967

[DMM052086C125] Nguyen, D., Jeon, H.-M. and Lee, J. (2022). Tissue factor links inflammation, thrombosis, and senescence in COVID-19. *Sci. Rep.* 12, 19842. 10.1038/s41598-022-23950-y 36400883 PMC9673213

[DMM052086C126] Nori, W. and Ghani Zghair, M. A. (2022). Omicron targets upper airways in pediatrics, elderly and unvaccinated population. *World J. Clin. Cases* 10, 12062-12065. 10.12998/wjcc.v10.i32.12062 36405264 PMC9669854

[DMM052086C127] Oladunni, F. S., Park, J.-G., Pino, P. A., Gonzalez, O., Akhter, A., Allué-Guardia, A., Olmo-Fontánez, A., Gautam, S., Garcia-Vilanova, A., Ye, C. et al. (2020). Lethality of SARS-CoV-2 infection in K18 human angiotensin-converting enzyme 2 transgenic mice. *Nat. Commun.* 11, 6122. 10.1038/s41467-020-19891-7 33257679 PMC7705712

[DMM052086C128] Oosthuizen, K., Steyn, E. C., Tucker, L., Ncube, I. V., Hardie, D. and Marais, S. (2021). SARS-CoV-2 encephalitis presenting as a clinical cerebellar syndrome: a case report. *Neurology* 97, 27-29. 10.1212/WNL.0000000000012051 33853896

[DMM052086C129] Ostergaard, L. (2021). SARS CoV-2 related microvascular damage and symptoms during and after COVID-19: Consequences of capillary transit-time changes, tissue hypoxia and inflammation. *Physiol. Rep.* 9, e14726. 10.14814/phy2.14726 33523608 PMC7849453

[DMM052086C130] Osterrieder, N., Bertzbach, L. D., Dietert, K., Abdelgawad, A., Vladimirova, D., Kunec, D., Hoffmann, D., Beer, M., Gruber, A. D. and Trimpert, J. (2020). Age-dependent progression of SARS-CoV-2 infection in Syrian hamsters. *Viruses* 12, 779. 10.3390/v12070779 32698441 PMC7412213

[DMM052086C131] Pal, M., Berhanu, G., Desalegn, C. and Kandi, V. (2020). Severe acute respiratory syndrome coronavirus-2 (SARS-CoV-2): an update. *Cureus* 12, e7423. 32337143 10.7759/cureus.7423 PMC7182166

[DMM052086C132] Pan, L., Mu, M., Yang, P., Sun, Y., Wang, R., Yan, J., Li, P., Hu, B., Wang, J., Hu, C. et al. (2020). Clinical characteristics of COVID-19 patients with digestive symptoms in Hubei, China: a descriptive, cross-sectional, multicenter study. *Am. J. Gastroenterol.* 115, 766-773. 10.14309/ajg.0000000000000620 32287140 PMC7172492

[DMM052086C133] Park, J. C. and Im, S.-H. (2020). Of men in mice: the development and application of a humanized gnotobiotic mouse model for microbiome therapeutics. *Exp. Mol. Med.* 52, 1383-1396. 10.1038/s12276-020-0473-2 32908211 PMC8080820

[DMM052086C134] Parker, A., Fonseca, S. and Carding, S. R. (2020). Gut microbes and metabolites as modulators of blood-brain barrier integrity and brain health. *Gut Microbes* 11, 135-157. 10.1080/19490976.2019.1638722 31368397 PMC7053956

[DMM052086C135] Parsons, P. E., Eisner, M. D., Thompson, B. T., Matthay, M. A., Ancukiewicz, M., Bernard, G. R. and Wheeler, A. P. (2005). Lower tidal volume ventilation and plasma cytokine markers of inflammation in patients with acute lung injury. *Crit. Care Med.* 33, 1-6; discussion 230-2. 10.1097/01.CCM.0000149854.61192.DC 15644641

[DMM052086C136] Patankar, J. V., Chiriac, M. T., Lehmann, M., Kühl, A. A., Atreya, R., Becker, C., Gonzalez-Acera, M., Schmitt, H., Gamez-Belmonte, R., Mahapatro, M. et al. (2021). Severe acute respiratory syndrome coronavirus 2 attachment receptor angiotensin-converting enzyme 2 is decreased in Crohn's disease and regulated by microbial and inflammatory signaling. *Gastroenterology* 160, 925-928.e4. 10.1053/j.gastro.2020.10.021 33075345 PMC7567698

[DMM052086C137] Patrì, A., Vargas, M., Buonanno, P., Annunziata, M. C., Russo, D., Staibano, S., Servillo, G. and Fabbrocini, G. (2021). From SARS-CoV-2 hematogenous spreading to endothelial dysfunction: clinical-histopathological study of cutaneous signs of COVID-19. *Diagn. Pathol.* 16, 16. 10.1186/s13000-021-01075-6 33632250 PMC7905980

[DMM052086C138] Peluso, M. J. and Deeks, S. G. (2022). Early clues regarding the pathogenesis of long-COVID. *Trends Immunol.* 43, 268-270. 10.1016/j.it.2022.02.008 35272932 PMC8901423

[DMM052086C140] Phetsouphanh, C., Darley, D. R., Wilson, D. B., Howe, A., Munier, C. M. L., Patel, S. K., Juno, J. A., Burrell, L. M., Kent, S. J., Dore, G. J. et al. (2022). Immunological dysfunction persists for 8 months following initial mild-to-moderate SARS-CoV-2 infection. *Nat. Immunol.* 23, 210-216. 10.1038/s41590-021-01113-x 35027728

[DMM052086C141] Philippens, I., Böszörményi, K. P., Wubben, J. A. M., Fagrouch, Z. C., Van Driel, N., Mayenburg, A. Q., Lozovagia, D., Roos, E., Schurink, B., Bugiani, M. et al. (2022). Brain inflammation and intracellular α-Synuclein aggregates in macaques after SARS-CoV-2 infection. *Viruses* 14, 776. 10.3390/v14040776 35458506 PMC9025893

[DMM052086C142] Pretorius, E., Vlok, M., Venter, C., Bezuidenhout, J. A., Laubscher, G. J., Steenkamp, J. and Kell, D. B. (2021). Persistent clotting protein pathology in Long COVID/Post-Acute Sequelae of COVID-19 (PASC) is accompanied by increased levels of antiplasmin. *Cardiovasc. Diabetol.* 20, 172. 10.1186/s12933-021-01359-7 34425843 PMC8381139

[DMM052086C143] Proal, A. D., Vanelzakker, M. B., Aleman, S., Bach, K., Boribong, B. P., Buggert, M., Cherry, S., Chertow, D. S., Davies, H. E., Dupont, C. L. et al. (2023). SARS-CoV-2 reservoir in post-acute sequelae of COVID-19 (PASC). *Nat. Immunol.* 24, 1616-1627. 10.1038/s41590-023-01601-2 37667052

[DMM052086C144] Qin, C., Wang, J., Wei, Q., She, M., Marasco, W. A., Jiang, H., Tu, X., Zhu, H., Ren, L., Gao, H. et al. (2005). An animal model of SARS produced by infection of Macaca mulatta with SARS coronavirus. *J. Pathol.* 206, 251-259. 10.1002/path.1769 15892035 PMC7167940

[DMM052086C145] Rabbani, M. Y., Rappaport, J. and Gupta, M. K. (2022). Activation of immune system may cause pathophysiological changes in the myocardium of SARS-CoV-2 infected monkey model. *Cells* 11, 611. 10.3390/cells11040611 35203260 PMC8869860

[DMM052086C146] Renn, M., Bartok, E., Zillinger, T., Hartmann, G. and Behrendt, R. (2021). Animal models of SARS-CoV-2 and COVID-19 for the development of prophylactic and therapeutic interventions. *Pharmacol. Ther.* 228, 107931. 10.1016/j.pharmthera.2021.107931 34171328 PMC8219947

[DMM052086C147] Rhea, E. M., Logsdon, A. F., Hansen, K. M., Williams, L. M., Reed, M. J., Baumann, K. K., Holden, S. J., Raber, J., Banks, W. A. and Erickson, M. A. (2021). The S1 protein of SARS-CoV-2 crosses the blood–brain barrier in mice. *Nat. Neurosci.* 24, 368-378. 10.1038/s41593-020-00771-8 33328624 PMC8793077

[DMM052086C148] Richard, M., Kok, A., De Meulder, D., Bestebroer, T. M., Lamers, M. M., Okba, N. M. A., Fentener Van Vlissingen, M., Rockx, B., Haagmans, B. L., Koopmans, M. P. G. et al. (2020). SARS-CoV-2 is transmitted via contact and via the air between ferrets. *Nat. Commun.* 11, 3496. 10.1038/s41467-020-17367-2 32641684 PMC7343828

[DMM052086C149] Rizvi, Z. A., Dalal, R., Sadhu, S., Binayke, A., Dandotiya, J., Kumar, Y., Shrivastava, T., Gupta, S. K., Aggarwal, S., Tripathy, M. R. et al. (2022). Golden Syrian hamster as a model to study cardiovascular complications associated with SARS-CoV-2 infection. *eLife* 11, e73522. 10.7554/eLife.73522 35014610 PMC8794466

[DMM052086C150] Rowe, T., Gao, G., Hogan, R. J., Crystal, R. G., Voss, T. G., Grant, R. L., Bell, P., Kobinger, G. P., Wivel, N. A. and Wilson, J. M. (2004). Macaque model for severe acute respiratory syndrome. *J. Virol.* 78, 11401-11404. 10.1128/JVI.78.20.11401-11404.2004 15452262 PMC521815

[DMM052086C151] Rutkai, I., Mayer, M. G., Hellmers, L. M., Ning, B., Huang, Z., Monjure, C. J., Coyne, C., Silvestri, R., Golden, N., Hensley, K. et al. (2022). Neuropathology and virus in brain of SARS-CoV-2 infected non-human primates. *Nat. Commun.* 13, 1745. 10.1038/s41467-022-29440-z 35365631 PMC8975902

[DMM052086C152] Salguero, F. J., White, A. D., Slack, G. S., Fotheringham, S. A., Bewley, K. R., Gooch, K. E., Longet, S., Humphries, H. E., Watson, R. J., Hunter, L. et al. (2021). Comparison of rhesus and cynomolgus macaques as an infection model for COVID-19. *Nat. Commun.* 12, 1260. 10.1038/s41467-021-21389-9 33627662 PMC7904795

[DMM052086C153] Savla, S. R., Prabhavalkar, K. S. and Bhatt, L. K. (2021). Cytokine storm associated coagulation complications in COVID-19 patients: pathogenesis and management. *Expert Rev. Anti Infect. Ther.* 19, 1397-1413. 10.1080/14787210.2021.1915129 33832398 PMC8074652

[DMM052086C154] Schlottau, K., Rissmann, M., Graaf, A., Schön, J., Sehl, J., Wylezich, C., Höper, D., Mettenleiter, T. C., Balkema-Buschmann, A., Harder, T. et al. (2020). SARS-CoV-2 in fruit bats, ferrets, pigs, and chickens: an experimental transmission study. *Lancet Microbe* 1, e218-e225. 10.1016/S2666-5247(20)30089-6 32838346 PMC7340389

[DMM052086C155] Seeßle, J., Waterboer, T., Hippchen, T., Simon, J., Kirchner, M., Lim, A., Müller, B. and Merle, U. (2022). Persistent symptoms in adult patients 1 year after coronavirus disease 2019 (COVID-19): a prospective cohort study. *Clin. Infect. Dis.* 74, 1191-1198. 10.1093/cid/ciab611 34223884 PMC8394862

[DMM052086C156] Seibert, B., Cáceres, C. J., Cardenas-Garcia, S., Carnaccini, S., Geiger, G., Rajao, D. S., Ottesen, E. and Perez, D. R. (2021). Mild and severe SARS-CoV-2 infection induces respiratory and intestinal microbiome changes in the K18-hACE2 transgenic mouse model. *Microbiol. Spectr.* 9, e0053621. 10.1128/Spectrum.00536-21 34378965 PMC8455067

[DMM052086C157] Seibert, B., Cáceres, C. J., Carnaccini, S., Cardenas-Garcia, S., Gay, L. C., Ortiz, L., Geiger, G., Rajao, D. S., Ottesen, E. and Perez, D. R. (2022). Pathobiology and dysbiosis of the respiratory and intestinal microbiota in 14 months old Golden Syrian hamsters infected with SARS-CoV-2. *PLoS Pathog.* 18, e1010734. 10.1371/journal.ppat.1010734 36279276 PMC9632924

[DMM052086C158] Selickman, J., Vrettou, C. S., Mentzelopoulos, S. D. and Marini, J. J. (2022). COVID-19-related ARDS: key mechanistic features and treatments. *J. Clin. Med.* 11, 4896. 10.3390/jcm11164896 36013135 PMC9410336

[DMM052086C159] Sencio, V., Machelart, A., Robil, C., Benech, N., Hoffmann, E., Galbert, C., Deryuter, L., Heumel, S., Hantute-Ghesquier, A., Flourens, A. et al. (2022). Alteration of the gut microbiota following SARS-CoV-2 infection correlates with disease severity in hamsters. *Gut Microbes* 14, 2018900. 10.1080/19490976.2021.2018900 34965194 PMC8726722

[DMM052086C160] Serban, D., Tribus, L. C., Vancea, G., Stoian, A. P., Dascalu, A. M., Suceveanu, A. I., Tanasescu, C., Costea, A. C., Tudosie, M. S., Tudor, C. et al. (2021). Acute mesenteric ischemia in COVID-19 patients. *J. Clin. Med.* 11, 200. 10.3390/jcm11010200 35011941 PMC8745985

[DMM052086C161] Shan, C., Yao, Y.-F., Yang, X.-L., Zhou, Y.-W., Gao, G., Peng, Y., Yang, L., Hu, X., Xiong, J., Jiang, R.-D. et al. (2020). Infection with novel coronavirus (SARS-CoV-2) causes pneumonia in Rhesus macaques. *Cell Res.* 30, 670-677. 10.1038/s41422-020-0364-z 32636454 PMC7364749

[DMM052086C162] Shang, J., Ye, G., Shi, K., Wan, Y., Luo, C., Aihara, H., Geng, Q., Auerbach, A. and Li, F. (2020). Structural basis of receptor recognition by SARS-CoV-2. *Nature* 581, 221-224. 10.1038/s41586-020-2179-y 32225175 PMC7328981

[DMM052086C163] Sherif, Z. A., Gomez, C. R., Connors, T. J., Henrich, T. J., Reeves, W. B. and Force, R. M. P. T. (2023). Pathogenic mechanisms of post-acute sequelae of SARS-CoV-2 infection (PASC). *eLife* 12, e86002. 10.7554/eLife.86002 36947108 PMC10032659

[DMM052086C164] Shi, S., Qin, M., Shen, B., Cai, Y., Liu, T., Yang, F., Gong, W., Liu, X., Liang, J., Zhao, Q. et al. (2020). Association of cardiac injury with mortality in hospitalized patients with COVID-19 in Wuhan, China. *JAMA Cardiol.* 5, 802-810. 10.1001/jamacardio.2020.0950 32211816 PMC7097841

[DMM052086C165] Siddiqi, H. K., Libby, P. and Ridker, P. M. (2021). COVID-19 - a vascular disease. *Trends Cardiovasc. Med.* 31, 1-5. 10.1016/j.tcm.2020.10.005 33068723 PMC7556303

[DMM052086C166] Silva, Y. P., Bernardi, A. and Frozza, R. L. (2020). The role of short-chain fatty acids from gut microbiota in gut-brain communication. *Front. Endocrinol. (Lausanne)* 11, 25. 10.3389/fendo.2020.00025 32082260 PMC7005631

[DMM052086C167] Sokol, H., Contreras, V., Maisonnasse, P., Desmons, A., Delache, B., Sencio, V., Machelart, A., Brisebarre, A., Humbert, L., Deryuter, L. et al. (2021). SARS-CoV-2 infection in nonhuman primates alters the composition and functional activity of the gut microbiota. *Gut Microbes* 13, 1-19. 10.1080/19490976.2021.1893113 PMC7951961 33685349

[DMM052086C168] Soliman, M. L., Combs, C. K. and Rosenberger, T. A. (2013). Modulation of inflammatory cytokines and mitogen-activated protein kinases by acetate in primary astrocytes. *J. Neuroimmune Pharmacol.* 8, 287-300. 10.1007/s11481-012-9426-4 23233245 PMC3587660

[DMM052086C169] Solomon, I. H., Normandin, E., Bhattacharyya, S., Mukerji, S. S., Keller, K., Ali, A. S., Adams, G., Hornick, J. L., Padera, R. F., Jr. and Sabeti, P. (2020). Neuropathological features of Covid-19. *N. Engl. J. Med.* 383, 989-992. 10.1056/NEJMc2019373 32530583 PMC7304421

[DMM052086C170] Song, E., Zhang, C., Israelow, B., Lu-Culligan, A., Prado, A. V., Skriabine, S., Lu, P., Weizman, O. E., Liu, F., Dai, Y. et al. (2021). Neuroinvasion of SARS-CoV-2 in human and mouse brain. *J. Exp. Med.* 218, e20202135. 10.1084/jem.20202135 33433624 PMC7808299

[DMM052086C171] Speranza, E., Purushotham, J. N., Port, J. R., Schwarz, B., Flagg, M., Williamson, B. N., Feldmann, F., Singh, M., Perez-Perez, L., Sturdevant, G. L. et al. (2022). Age-related differences in immune dynamics during SARS-CoV-2 infection in rhesus macaques. *Life Sci. Alliance* 5, e202101314. 10.26508/lsa.202101314 35039442 PMC8807873

[DMM052086C172] Stein, S. R., Ramelli, S. C., Grazioli, A., Chung, J.-Y., Singh, M., Yinda, C. K., Winkler, C. W., Sun, J., Dickey, J. M., Ylaya, K. et al. (2022). SARS-CoV-2 infection and persistence in the human body and brain at autopsy. *Nature* 612, 758-763. 10.1038/s41586-022-05542-y 36517603 PMC9749650

[DMM052086C173] Stokes, E. K., Zambrano, L. D., Anderson, K. N., Marder, E. P., Raz, K. M., El Burai Felix, S., Tie, Y. and Fullerton, K. E. (2020). Coronavirus disease 2019 case surveillance - United States, January 22-May 30, (2020). *MMWR Morb. Mortal. Wkly. Rep.* 69, 759-765. 10.15585/mmwr.mm6924e2 32555134 PMC7302472

[DMM052086C174] Su, Y., Yuan, D., Chen, D. G., Ng, R. H., Wang, K., Choi, J., Li, S., Hong, S., Zhang, R., Xie, J. et al. (2022). Multiple early factors anticipate post-acute COVID-19 sequelae. *Cell* 185, 881-895.e20. 10.1016/j.cell.2022.01.014 35216672 PMC8786632

[DMM052086C175] Subramanian, A., Nirantharakumar, K., Hughes, S., Myles, P., Williams, T., Gokhale, K. M., Taverner, T., Chandan, J. S., Brown, K., Simms-Williams, N. et al. (2022). Symptoms and risk factors for long COVID in non-hospitalized adults. *Nat. Med.* 28, 1706-1714. 10.1038/s41591-022-01909-w 35879616 PMC9388369

[DMM052086C176] Sultan, S., Altayar, O., Siddique, S. M., Davitkov, P., Feuerstein, J. D., Lim, J. K., Falck-Ytter, Y. and El-Serag, H. B. (2020). AGA institute rapid review of the gastrointestinal and liver manifestations of COVID-19, meta-analysis of international data, and recommendations for the consultative management of patients with COVID-19. *Gastroenterology* 159, 320-334.e27. 10.1053/j.gastro.2020.05.001 32407808 PMC7212965

[DMM052086C177] Szarpak, L., Mierzejewska, M., Jurek, J., Kochanowska, A., Gasecka, A., Truszewski, Z., Pruc, M., Blek, N., Rafique, Z., Filipiak, K. J. et al. (2022). Effect of coronary artery disease on COVID-19-prognosis and risk assessment: a systematic review and meta-analysis. *Biology (Basel)* 11, 221. 10.3390/biology11020221 35205088 PMC8868600

[DMM052086C178] Tahyra, A. S. C., Calado, R. T. and Almeida, F. (2022). The Role of Extracellular Vesicles in COVID-19 Pathology. *Cells* 11, 2496. 10.3390/cells11162496 36010572 PMC9406571

[DMM052086C179] Thaweethai, T., Jolley, S. E., Karlson, E. W., Levitan, E. B., Levy, B., McComsey, G. A., McCorkell, L., Nadkarni, G. N., Parthasarathy, S., Singh, U. et al. (2023). Development of a definition of postacute sequelae of SARS-CoV-2 infection. *JAMA* 329, 1934-1946. 10.1001/jama.2023.8823 37278994 PMC10214179

[DMM052086C180] To, E. E., Vlahos, R., Luong, R., Halls, M. L., Reading, P. C., King, P. T., Chan, C., Drummond, G. R., Sobey, C. G., Broughton, B. R. S. et al. (2017). Endosomal NOX2 oxidase exacerbates virus pathogenicity and is a target for antiviral therapy. *Nat. Commun.* 8, 69. 10.1038/s41467-017-00057-x 28701733 PMC5507984

[DMM052086C181] Trevino, T. N., Almousawi, A. A., Robinson, K. F., Fogel, A. B., Class, J., Minshall, R. D., Tai, L. M., Richner, J. M. and Lutz, S. E. (2024). Caveolin-1 mediates blood-brain barrier permeability, neuroinflammation, and cognitive impairment in SARS-CoV-2 infection. *J. Neuroimmunol.* 388, 578309. 10.1016/j.jneuroim.2024.578309 38335781 PMC11212674

[DMM052086C182] Trimpert, J., Vladimirova, D., Dietert, K., Abdelgawad, A., Kunec, D., Dökel, S., Voss, A., Gruber, A. D., Bertzbach, L. D. and Osterrieder, N. (2020). The Roborovski Dwarf hamster is a highly susceptible model for a rapid and fatal course of SARS-CoV-2 infection. *Cell Rep.* 33, 108488. 10.1016/j.celrep.2020.108488 33271063 PMC7674129

[DMM052086C183] Urano, E., Okamura, T., Ono, C., Ueno, S., Nagata, S., Kamada, H., Higuchi, M., Furukawa, M., Kamitani, W., Matsuura, Y. et al. (2021). COVID-19 cynomolgus macaque model reflecting human COVID-19 pathological conditions. *Proc. Natl. Acad. Sci. USA* 118, e2104847118. 10.1073/pnas.2104847118 34625475 PMC8639365

[DMM052086C184] Usai, C., Mateu, L., Brander, C., Vergara-Alert, J. and Segalés, J. (2023). Animal models to study the neurological manifestations of the post-COVID-19 condition. *Lab. Anim. (NY)* 52, 202-210. 10.1038/s41684-023-01231-z 37620562 PMC10462483

[DMM052086C185] Vallender, E. J. and Miller, G. M. (2013). Nonhuman primate models in the genomic era: a paradigm shift. *ILAR J.* 54, 154-165. 10.1093/ilar/ilt044 24174439 PMC3814397

[DMM052086C186] Van De Ven, K., Van Dijken, H., Wijsman, L., Gomersbach, A., Schouten, T., Kool, J., Lenz, S., Roholl, P., Meijer, A., Van Kasteren, P. B. et al. (2021). Pathology and immunity after SARS-CoV-2 infection in male ferrets is affected by age and inoculation route. *Front. Immunol.* 12, 750229. 10.3389/fimmu.2021.750229 34745122 PMC8566349

[DMM052086C187] Van Riel, D., Munster, V. J., De Wit, E., Rimmelzwaan, G. F., Fouchier, R. A., Osterhaus, A. D. and Kuiken, T. (2007). Human and avian influenza viruses target different cells in the lower respiratory tract of humans and other mammals. *Am. J. Pathol.* 171, 1215-1223. 10.2353/ajpath.2007.070248 17717141 PMC1988871

[DMM052086C188] Vanderheiden, A., Hill, J. D., Jiang, X., Deppen, B., Bamunuarachchi, G., Soudani, N., Joshi, A., Cain, M. D., Boon, A. C. M. and Klein, R. S. (2024). Vaccination reduces central nervous system IL-1β and memory deficits after COVID-19 in mice. *Nat. Immunol.* 25, 1158-1171. 10.1038/s41590-024-01868-z 38902519 PMC13148132

[DMM052086C189] Varatharaj, A., Thomas, N., Ellul, M. A., Davies, N. W. S., Pollak, T. A., Tenorio, E. L., Sultan, M., Easton, A., Breen, G., Zandi, M. et al. (2020). Neurological and neuropsychiatric complications of COVID-19 in 153 patients: a UK-wide surveillance study. *Lancet Psychiatry* 7, 875-882. 10.1016/S2215-0366(20)30287-X 32593341 PMC7316461

[DMM052086C190] Vidal, E., López-Figueroa, C., Rodon, J., Pérez, M., Brustolin, M., Cantero, G., Guallar, V., Izquierdo-Useros, N., Carrillo, J., Blanco, J. et al. (2022). Chronological brain lesions after SARS-CoV-2 infection in hACE2-transgenic mice. *Vet. Pathol.* 59, 613-626. 10.1177/03009858211066841 34955064 PMC9207990

[DMM052086C191] Violi, F., Oliva, A., Cangemi, R., Ceccarelli, G., Pignatelli, P., Carnevale, R., Cammisotto, V., Lichtner, M., Alessandri, F., De Angelis, M. et al. (2020). Nox2 activation in Covid-19. *Redox Biol.* 36, 101655. 10.1016/j.redox.2020.101655 32738789 PMC7381406

[DMM052086C192] V'Kovski, P., Kratzel, A., Steiner, S., Stalder, H. and Thiel, V. (2021). Coronavirus biology and replication: implications for SARS-CoV-2. *Nat. Rev. Microbiol.* 19, 155-170. 10.1038/s41579-020-00468-6 33116300 PMC7592455

[DMM052086C193] Von Weyhern, C. H., Kaufmann, I., Neff, F. and Kremer, M. (2020). Early evidence of pronounced brain involvement in fatal COVID-19 outcomes. *Lancet* 395, e109. 10.1016/S0140-6736(20)31282-4 32505222 PMC7272176

[DMM052086C194] Wang, H. and Ma, S. (2008). The cytokine storm and factors determining the sequence and severity of organ dysfunction in multiple organ dysfunction syndrome. *Am. J. Emerg. Med.* 26, 711-715. 10.1016/j.ajem.2007.10.031 18606328

[DMM052086C195] Wang, J., Chen, S. and Bihl, J. (2020). Exosome-mediated transfer of ACE2 (angiotensin-converting enzyme 2) from endothelial progenitor cells promotes survival and function of endothelial cell. *Oxid. Med. Cell Longev.* 2020, 4213541. 32051731 10.1155/2020/4213541 PMC6995312

[DMM052086C196] Wiertsema, S. P., Van Bergenhenegouwen, J., Garssen, J. and Knippels, L. M. J. (2021). The interplay between the gut microbiome and the immune system in the context of infectious diseases throughout life and the role of nutrition in optimizing treatment strategies. *Nutrients* 13, 886. 10.3390/nu13030886 33803407 PMC8001875

[DMM052086C197] Willemsen, L. E., Koetsier, M. A., Van Deventer, S. J. and Van Tol, E. A. (2003). Short chain fatty acids stimulate epithelial mucin 2 expression through differential effects on prostaglandin E(1) and E(2) production by intestinal myofibroblasts. *Gut* 52, 1442-1447. 10.1136/gut.52.10.1442 12970137 PMC1773837

[DMM052086C198] Winkler, E. S., Bailey, A. L., Kafai, N. M., Nair, S., McCune, B. T., Yu, J., Fox, J. M., Chen, R. E., Earnest, J. T., Keeler, S. P. et al. (2020). SARS-CoV-2 infection of human ACE2-transgenic mice causes severe lung inflammation and impaired function. *Nat. Immunol.* 21, 1327-1335. 10.1038/s41590-020-0778-2 32839612 PMC7578095

[DMM052086C199] Woolsey, C., Borisevich, V., Prasad, A. N., Agans, K. N., Deer, D. J., Dobias, N. S., Heymann, J. C., Foster, S. L., Levine, C. B., Medina, L. et al. (2021). Establishment of an African green monkey model for COVID-19 and protection against re-infection. *Nat. Immunol.* 22, 86-98. 10.1038/s41590-020-00835-8 33235385 PMC7790436

[DMM052086C200] Wu, X., Jing, H., Wang, C., Wang, Y., Zuo, N., Jiang, T., Novakovic, V. A. and Shi, J. (2022). Intestinal damage in COVID-19: SARS-CoV-2 infection and intestinal thrombosis. *Front. Microbiol.* 13, 860931. 10.3389/fmicb.2022.860931 35391725 PMC8981312

[DMM052086C201] Xiao, F., Tang, M., Zheng, X., Liu, Y., Li, X. and Shan, H. (2020). Evidence for gastrointestinal infection of SARS-CoV-2. *Gastroenterology* 158, 1831-1833.e3. 10.1053/j.gastro.2020.02.055 32142773 PMC7130181

[DMM052086C202] Xie, Y., Xu, E., Bowe, B. and Al-Aly, Z. (2022). Long-term cardiovascular outcomes of COVID-19. *Nat. Med.* 28, 583-590. 10.1038/s41591-022-01689-3 35132265 PMC8938267

[DMM052086C203] Xue, Y., Yang, D., Vogel, P., Stabenow, J., Zalduondo, L., Kong, Y., Ravi, Y., Sai-Sudhakar, C. B., Parvathareddy, J., Hayes, E. et al. (2022). Cardiopulmonary injury in the Syrian hamster model of COVID-19. *Viruses* 14, 1403. 10.3390/v14071403 35891384 PMC9316644

[DMM052086C204] Xydakis, M. S., Dehgani-Mobaraki, P., Holbrook, E. H., Geisthoff, U. W., Bauer, C., Hautefort, C., Herman, P., Manley, G. T., Lyon, D. M. and Hopkins, C. (2020). Smell and taste dysfunction in patients with COVID-19. *Lancet Infect. Dis.* 20, 1015-1016. 10.1016/S1473-3099(20)30293-0 32304629 PMC7159875

[DMM052086C205] Yaghi, S., Ishida, K., Torres, J., Mac Grory, B., Raz, E., Humbert, K., Henninger, N., Trivedi, T., Lillemoe, K., Alam, S. et al. (2020). SARS-CoV-2 and stroke in a New York healthcare system. *Stroke* 51, 2002-2011. 10.1161/STROKEAHA.120.030335 32432996 PMC7258764

[DMM052086C206] Yang, L. L., Millischer, V., Rodin, S., Macfabe, D. F., Villaescusa, J. C. and Lavebratt, C. (2020). Enteric short-chain fatty acids promote proliferation of human neural progenitor cells. *J. Neurochem.* 154, 635-646. 10.1111/jnc.14928 31784978

[DMM052086C207] Yao, Y., Bao, L., Deng, W., Xu, L., Li, F., Lv, Q., Yu, P., Chen, T., Xu, Y., Zhu, H. et al. (2014). An animal model of MERS produced by infection of rhesus macaques with MERS coronavirus. *J. Infect. Dis.* 209, 236-242. 10.1093/infdis/jit590 24218506 PMC7107340

[DMM052086C208] Ye, Q., Wang, B. and Mao, J. (2020). The pathogenesis and treatment of the ′Cytokine Storm’ in COVID-19. *J. Infect.* 80, 607-613. 10.1016/j.jinf.2020.03.037 32283152 PMC7194613

[DMM052086C209] Ye, Q., Zhou, J., He, Q., Li, R.-T., Yang, G., Zhang, Y., Wu, S.-J., Chen, Q., Shi, J.-H., Zhang, R.-R. et al. (2021). SARS-CoV-2 infection in the mouse olfactory system. *Cell Discov.* 7, 49. 10.1038/s41421-021-00290-1 34230457 PMC8260584

[DMM052086C210] Yeoh, Y. K., Zuo, T., Lui, G. C., Zhang, F., Liu, Q., Li, A. Y., Chung, A. C., Cheung, C. P., Tso, E. Y., Fung, K. S. et al. (2021). Gut microbiota composition reflects disease severity and dysfunctional immune responses in patients with COVID-19. *Gut* 70, 698-706. 10.1136/gutjnl-2020-323020 33431578 PMC7804842

[DMM052086C211] Yin, K., Peluso, M. J., Luo, X., Thomas, R., Shin, M.-G., Neidleman, J., Andrew, A., Young, K. C., Ma, T., Hoh, R. et al. (2024). Long COVID manifests with T cell dysregulation, inflammation and an uncoordinated adaptive immune response to SARS-CoV-2. *Nat. Immunol.* 25, 218-225. 10.1038/s41590-023-01724-6 38212464 PMC10834368

[DMM052086C212] Yinda, C. K., Port, J. R., Bushmaker, T., Offei Owusu, I., Purushotham, J. N., Avanzato, V. A., Fischer, R. J., Schulz, J. E., Holbrook, M. G., Hebner, M. J. et al. (2021). K18-hACE2 mice develop respiratory disease resembling severe COVID-19. *PLoS Pathog.* 17, e1009195. 10.1371/journal.ppat.1009195 33465158 PMC7875348

[DMM052086C213] Zhang, H., Li, H. B., Lyu, J. R., Lei, X. M., Li, W., Wu, G., Lyu, J. and Dai, Z. M. (2020). Specific ACE2 expression in small intestinal enterocytes may cause gastrointestinal symptoms and injury after 2019-nCoV infection. *Int. J. Infect. Dis.* 96, 19-24. 10.1016/j.ijid.2020.04.027 32311451 PMC7165079

[DMM052086C214] Zhao, X., Chen, D., Szabla, R., Zheng, M., Li, G., Du, P., Zheng, S., Li, X., Song, C., Li, R. et al. (2020). Broad and differential animal angiotensin-converting enzyme 2 receptor usage by SARS-CoV-2. *J. Virol.* 94, e00940-20. 10.1128/JVI.00940-20 32661139 PMC7459545

[DMM052086C215] Zheng, H., Li, H., Guo, L., Liang, Y., Li, J., Wang, X., Hu, Y., Wang, L., Liao, Y., Yang, F. et al. (2020). Virulence and pathogenesis of SARS-CoV-2 infection in rhesus macaques: A nonhuman primate model of COVID-19 progression. *PLoS Pathog.* 16, e1008949. 10.1371/journal.ppat.1008949 33180882 PMC7660522

[DMM052086C216] Zheng, J., Wong, L. R., Li, K., Verma, A. K., Ortiz, M. E., Wohlford-Lenane, C., Leidinger, M. R., Knudson, C. M., Meyerholz, D. K., McCray, P. B. Jr et al. (2021). COVID-19 treatments and pathogenesis including anosmia in K18-hACE2 mice. *Nature* 589, 603-607. 10.1038/s41586-020-2943-z 33166988 PMC7855185

[DMM052086C217] Zipeto, D., Palmeira, J. D. F., Argañaraz, G. A. and Argañaraz, E. R. (2020). ACE2/ADAM17/TMPRSS2 interplay may be the main risk factor for COVID-19. *Front. Immunol.* 11, 576745. 10.3389/fimmu.2020.576745 33117379 PMC7575774

[DMM052086C218] Zuo, T., Zhang, F., Lui, G. C. Y., Yeoh, Y. K., Li, A. Y. L., Zhan, H., Wan, Y., Chung, A. C. K., Cheung, C. P., Chen, N. et al. (2020). Alterations in gut microbiota of patients with COVID-19 during time of hospitalization. *Gastroenterology* 159, 944-955.e8. 10.1053/j.gastro.2020.05.048 32442562 PMC7237927

